# Protein oxidation in crowded environments

**DOI:** 10.1042/BCJ20250150

**Published:** 2026-04-02

**Authors:** Eduardo Fuentes-Lemus

**Affiliations:** Departamento de Química Física, Escuela de Química, Facultad de Química y de Farmacia, Pontificia Universidad Católica de Chile, Santiago, Chile

**Keywords:** macromolecular crowding, oxidative stress, post translational modification, protein confinement, Protein oxidation, redox signalling

## Abstract

Proteins are the most abundant macromolecules in biological systems. This high abundance and the presence of electron-rich side-chains make proteins a major target for biological oxidants. Protein oxidation encompasses a complex set of reactions that, depending on protein structure and the chemical properties of the oxidant, can trigger specific and reversible modifications, or can irreversibly damage multiple side-chains. Therefore, understanding protein oxidation from a mechanistic and kinetic perspective is important to illuminate the molecular basis of physiological (e.g. redox signaling) and pathological processes (e.g. cardiovascular disease and neurodegenerative diseases). However, an existing conundrum in the redox biochemistry field is whether (and how) intrinsic properties of biological environments, such as the crowded intracellular conditions resulting from the high abundance of macromolecules and protein confinement, modulate oxidation rates and pathways. These obvious, but often neglected, aspects of biological environments have begun to be systematically addressed, suggesting that the crowded intracellular conditions would be an important player in the oxidative biology of proteins. This review outlines the importance of protein oxidation in physiology and pathology. Then, thoroughly discusses the modulatory effect that crowding exerts on biochemical processes that involve proteins, particularly on the oxidative modification of proteins. Finally, evidence that illustrates the interplay that would exist between crowding, protein oxidation, and protein confinement by phase separation is discussed. The author proposes that the transition from using dilute *in vitro* studies to an experimental workflow that takes into account the crowded and heterogeneous conditions encountered is the cell is mandatory to rigorously investigate protein oxidation.

## Introduction

Oxidants generated in biological milieus, including both free radicals and two-electron oxidants, play important roles in cell physiology and pathology (reviewed in [1–3]). Due to their electrophilic chemical nature, these species can react with a variety of electron-rich molecules in biological systems, including proteins, lipids and nucleic acids [4–7]. However, the reactivity of biomolecules with oxidants can be vastly different, with reported rate constants ranging from *k* < 0.1 M^−1^ s^−1^ to those limited by the diffusion of reactants (i.e. *k* > 10^9^ M^−1^ s^−1^). Amongst biological macromolecules, proteins have been identified as important targets of reactive species. These reactions result in the modification of sidechains, which, depending on the modification site and chemical nature of the modification, can have a major impact on the protein structure and function [7–9]. The fact that proteins are major targets of oxidants in the biological context is sustained by their high abundance as these constitute roughly half of the cell dry mass with an estimated abundance of billions of proteins per cell [10], and the presence of electron-rich sidechains including the sulfur-containing amino acids cysteine (Cys) and methionine (Met), as well as, aromatic residues. Yet, despite proteomic studies revealing that an important portion of the proteome is susceptible to oxidative modifications with nearly 20% of Cys-containing proteins being modified [11], physiological redox signaling is governed by the specificity, sensitivity and function of the targeted protein [3]. Out of a total proteome estimated to be ∼20,000 without considering the many existing proteoforms generated by enzymatic and non-enzymatic post-translational modifications [12], the biological outcomes of oxidative modifications remain highly dependent on the target protein and the structure–function relationship. Thus, a major task in the redox biology and redox (bio)chemistry fields has been to illuminate the structural and spatiotemporal factors that make certain proteins susceptible to undergo oxidative modifications under physiological conditions and how these oxidative modifications are then transduced into signals that contribute to cell adaptation, communication, survival, and proliferation. In this context, an existing conundrum that is starting to be systematically addressed is whether (and how) intrinsic properties of biological environments such as confinement, microdomains, buffer composition, and/or the crowded intracellular conditions resulting from the high abundance of macromolecules (c.f. up to 40% of the intracellular volume is occupied by macromolecules [13]) modulate protein oxidation [13–16]. This invited review article will discuss the existing literature on the effect of macromolecular crowding, an obvious, but often neglected characteristic of biological environments, on the oxidative biology of proteins, with a critical vision on the implications of this for the redox biochemistry and redox biology fields. Firstly, an overview of the oxidative biology of proteins will be presented, with a focus on chemical modifications to protein sidechains including reaction rates and kinetic data, and the importance of the oxidative modification of proteins in physiology and pathology. Subsequently, it will be briefly discussed how crowded systems modulate different aspects of protein biophysics and biochemistry, thus having a major impact on reactions and interactions involving proteins with other (macro)molecules (including oxidants), to then discuss works addressing the effects that macromolecular crowding may have on protein oxidation. Finally, the link between crowding, protein oxidation and confinement will be addressed before concluding this review article with methodological approaches that can be used to rigorously investigate protein oxidation in crowded and heterogeneous systems, and a brief discussion of the outlook for this developing field.

## The oxidative modification of proteins in physiology and pathology

Proteins are called the workhorses of cells for the numerous functions they fulfil (spanning from enzymes and structural support to signaling, transport, and immune function). Thus, it is not surprising that modifications to their sidechains does not only impact their structure but can also have major consequences at a functional level. Yet, the oxidative biology of proteins is very complex and is difficult to predict universal consequences for oxidative reactions and modifications. In fact, the type and extent of damage to proteins depends on a range of factors, including:

1. Chemical nature of the oxidant molecule → highly reactive radicals (e.g. hydroxyl radical, ^•^OH) can oxidize most sidechains and the backbone with second order rate constants in the range of 10^7^–10^10^ M^−1^ s^−1^, whilst, other reactive species such as hydrogen peroxide (H_2_O_2_) show a greater selectivity with regard to the residues targeted (Table 1) [7,17,18].
2. Abundance of the target protein → the intracellular concentration of proteins can vary from very low to very high, spanning from the low nanomolar to the millimolar concentration range.
3. The microenvironment where the target residue is located → solvent accessibility dictates an important difference in terms of oxidation rates of buried versus solvent-accessible residues. Additionally, other (local) structural factors inherent to specific proteins, such as the presence of residues that increase the acidity of the thiol moiety of Cys (increasing its nucleophilicity), makes these more reactive compared with most protein thiols (see Table 2).
4. The products formed → reversible versus irreversible products, the occurrence of secondary reactions, including chain reactions (*vide infra*) [19,20].
5. Intra- and inter-molecular transfer reactions [21].
6. Intrinsic properties of the milieu including buffer composition, local viscosities and macromolecular crowding [13].

Thus, while reversible modifications at specific residues may play important roles for biological processes, accumulation of oxidative damage, and/or formation of irreversible products including protein cross-linking or fragmentation of the backbone can be a cause, or a result, of dysfunction and pathology including cardiometabolic and neurodegenerative diseases, inflammation, and cancer, amongst others [22–25].

**Table 1:** Apparent second-order rate constant for the reaction of ^•^OH, or H_2_O_2_, with free amino acids at pH 7.0 and room temperature (∼20°C)

Oxidant	Target	Second-order rate constant (M^−1^ s^−1^)	Reference
^•^OH	Cys	3.4 × 10^10^	[7]
	Met	8.3 × 10^9^	
	Aromatic amino acids (Phe, Tyr, Trp)	10^9^–10^10^	
	Aliphatic amino acids (Gly, Ala, Val, Leu, Ile, Pro)	10^7^–10^9^	
	Polar amino acids (Ser, Thr, Gln, Asn)	10^7^–10^8^	
	Basic amino acids (His, Lys, Arg)	10^8^–10^10^	
	Acid amino acids (Asp, Glu)	10^7^–10^8^	
H_2_O_2_	Cys	0.84	[26]
	Met	7 × 10^−3^	[18]
	Other amino acids	Unreactive, unless in the presence of transition metals	

**Table 2:** Apparent second-order rate constants for reaction of Cys with H_2_O_2_ in different protein environments at pH 7 and room temperature (∼20°C)

Target	Second-order rate constant (M^−1^ s^−1^)	Reference
Cys	0.84	[26]
GSH	0.89	
Cys34 in BSA	2.3	[27]
Catalytic Cys in GAPDH (152 in the human isoform)	7	[28]
Catalytic Cys in PTP1B	9.1	[29]
Catalytic Cys in Prdx1 (cytosolic)	3.8 × 10^7^	[30]
Catalytic Cys in Prdx2 (cytosolic)	1 × 10^8^	
Catalytic Cys in Prdx3 (mitochondrial)	2 × 10^7^	

*GSH: glutathione; GAPDH: glyceraldehyde-3-phosphate dehydrogenase; PTP1B: Protein tyrosine phosphatase 1B; Prdx: peroxiredoxins.

### Protein oxidation in redox signaling

**Figure 1:**
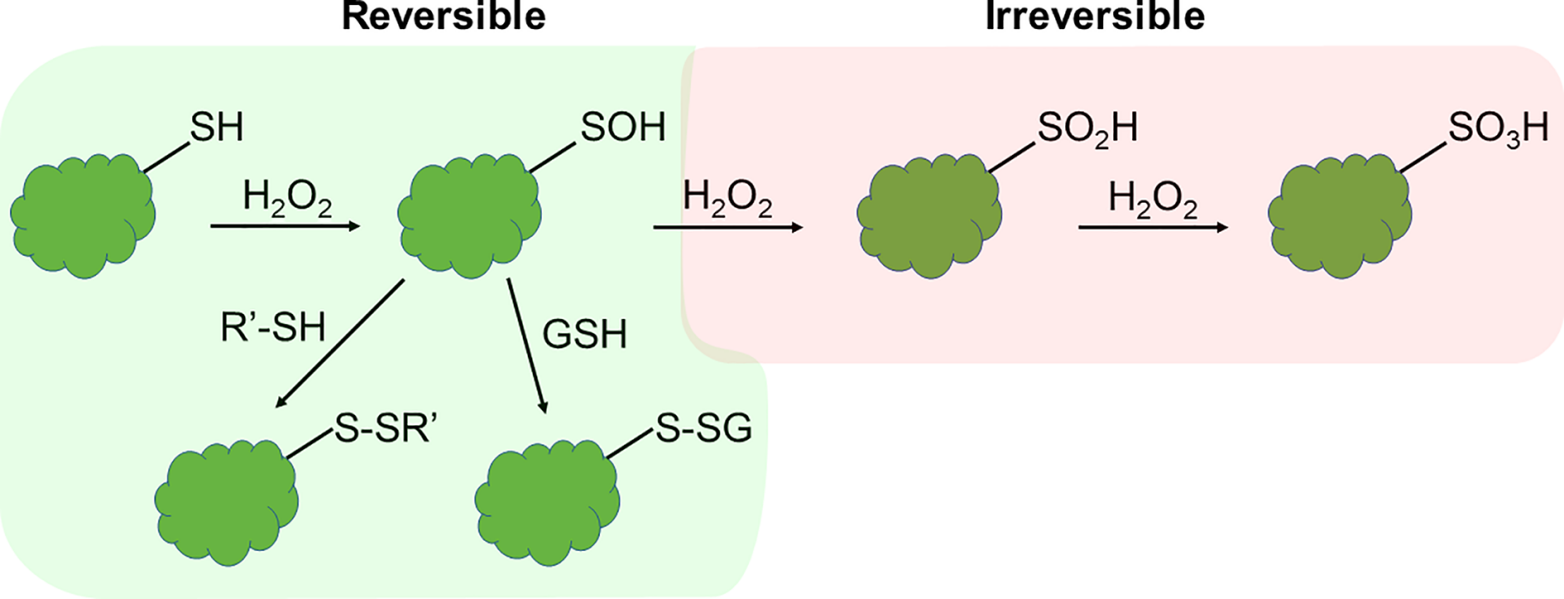
Oxidative modification of protein thiols (Cys) by physiological (green box) and supraphysiological (red box) hydrogen peroxide (H_2_O_2_) levels The initial reaction product corresponds to a sulfenic acid (Cys-SOH). Cys-SOH can react with a second Cys to yield intra- or intermolecular disulfides, or it can react with glutathione (GSH) leading to the formation of glutathionylated proteins. These products can be reduced by the thioredoxin/thioredoxin reductase, or the GSH/glutaredoxin systems (*vide infra*, Figure 2) to regenerate the reduced Cys. In the presence of an excess of H_2_O_2_, Cys-SOH can be oxidized to the oxyacids sulfinic (Cys-SO_2_H) and sulfonic (Cys-SO_3_H) acids, which are irreversible products.

**Figure 2:**
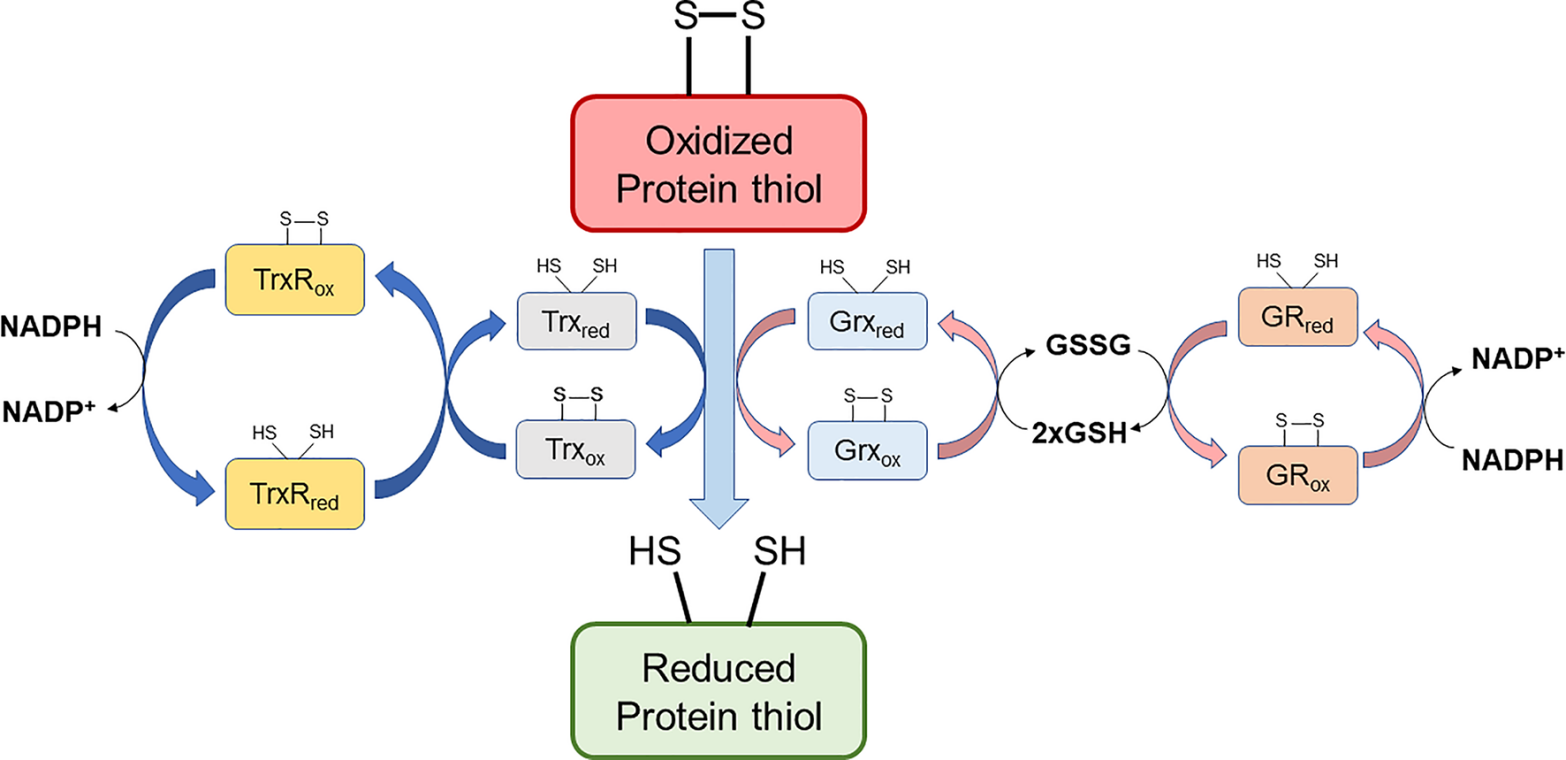
Scheme of oxidoreductase activities that allow that protein thiol oxidation is resolvable allowing redox signaling Reduced nicotinamide adenine dinucleotide phosphate (NADPH) is generated by enzymes of the oxidative branch of the pentose phosphate pathway, and to a minor extent by the malic enzyme and isocitrate dehydrogenase. NADPH reduces oxidized thioredoxin reductase (TrxR), and oxidized glutathione reductase (GR). These systems contribute to redox cycling via the thioredoxin (Trx), or the glutaredoxin (Grx) systems; GSH, glutathione; GSSG, glutathione disulfide.

The concept of protein oxidation has undergone a significant paradigm shift over the last decades thanks to the rigorous work and contributions by the international redox community [1,31–33]. Thus, the perception that oxidative modifications were purely damaging has shifted now that it is known that under physiological conditions the important biological oxidant molecule H_2_O_2_ is maintained within low (steady-state) levels inducing the specific and reversible chemical modification of target proteins [1,34]. In the absence of transition metals, the ionized thiolate anion form of Cys (Cys-S^−^) is by far the most reactive amino acid toward H_2_O_2_ and other hydroperoxides. Hence, the nucleophilicity and reactivity of the thiol moiety is dramatically influenced by the microenvironment where Cys residues sit within the protein structure [17,35]. The initial oxidation of Cys leads to the formation of a sulfenic acid (Cys-SOH) (Figure 1). Then, this transient intermediate can be converted into stable, reversible, post-translational modifications that switch protein function “on” or “off.” The latter includes the formation of intra- or intermolecular disulfide bonds (R-S-S-R’) or S-glutathionylation. These precise modifications can be rapidly reversed by dedicated cellular systems, such as thioredoxin and glutaredoxin pathways, ensuring the signal is transient and resolvable (Figure 2) [36–38]. Further oxidation of the sulfenic acids leads to the formation of sulfinic acid which was historically considered an irreversible product, yet, recent reports have shown the existence of a dedicated family of reductases capable of reducing these species in peroxiredoxins and other proteins [39–41]. By acting as a functional “redox switch,” protein oxidation controls crucial signaling pathways. For instance, the oxidation of specific Cys residues can regulate the activity of key signaling/metabolic nodes like peroxiredoxins, glyceraldehyde-3-phosphate dehydrogenase (GAPDH), protein tyrosine phosphatases (PTPs), and various protein kinases [29,42–48]. In addition to these well-characterized redox regulatory mechanisms involving Cys, another residue that is also susceptible to oxidation by H_2_O_2_ is Met, although with a significantly slower reaction rate than Cys (Table 1). Met oxidation leads to the formation of methionine sulfoxide (MetSO) [49,50]. MetSO has a profound impact at protein structure level as it changes the polarity of Met altering protein conformation and function [50]. Despite being a stable and long-lived product, MetSO can be reduced *in vivo* by a specialized family of reductases (MetSO reductases [51]), allowing this oxidative modification to also play an important role in redox signaling pathways. For example, it has been described that oxidation of specific Met residues can have a negative impact on protein phosphorylation, as reported for the mitochondrial pyruvate dehydrogenase E1α subunit [52], α-synuclein [53], and on the inhibitor protein of the NF-κB transcription factor IκBα protein [54]. Met oxidation to its reversible product has also been reported to affect other signaling pathways including calcium homeostasis, mitochondrial function and glucose regulation as recently reviewed [55]. In summary, the reversibility and site-specificity of the oxidative modification of proteins distinguish redox signaling from non-specific damage, highlighting its role as an essential, evolved mechanism for rapid cellular adaptation and communication in response to both endogenous metabolic shifts and external stimuli.

### Accumulation of oxidative damage on proteins

Contrary to the site-specific and reversible oxidative modifications discussed above, the accumulation of oxidative damage, or the formation of irreversible products, can significantly and permanently alter protein structure and function. Yet, not all proteins are equal in terms of robustness/susceptibility against oxidative damage, with some oxidized proteins partially preserving their function upon oxidation, whilst others can be easily inactivated by oxidation [56–59]. Furthermore, moderate protein oxidation also serves a physiological role. It has been proposed to be an important player in protein turnover and a marker for protein degradation by proteases (e.g. 20S proteasome and lysosomal activity in the cytosol, and Lon and Clp proteases in the mitochondria) [60–63]. Contrarily, massive oxidative damage results in decreased turnover [61–69]. This has been associated with the accumulation of oxidized proteins in multiple human pathologies, including neurodegenerative diseases (e.g. Alzheimer’s disease and Parkinson’s disease), cataractogenesis, and cardiometabolic diseases, amongst other pathologies associated with increased oxidative status [22,25,70]. Accumulation of damaged proteins has also been linked to aging [71–75].

**Figure 3:**
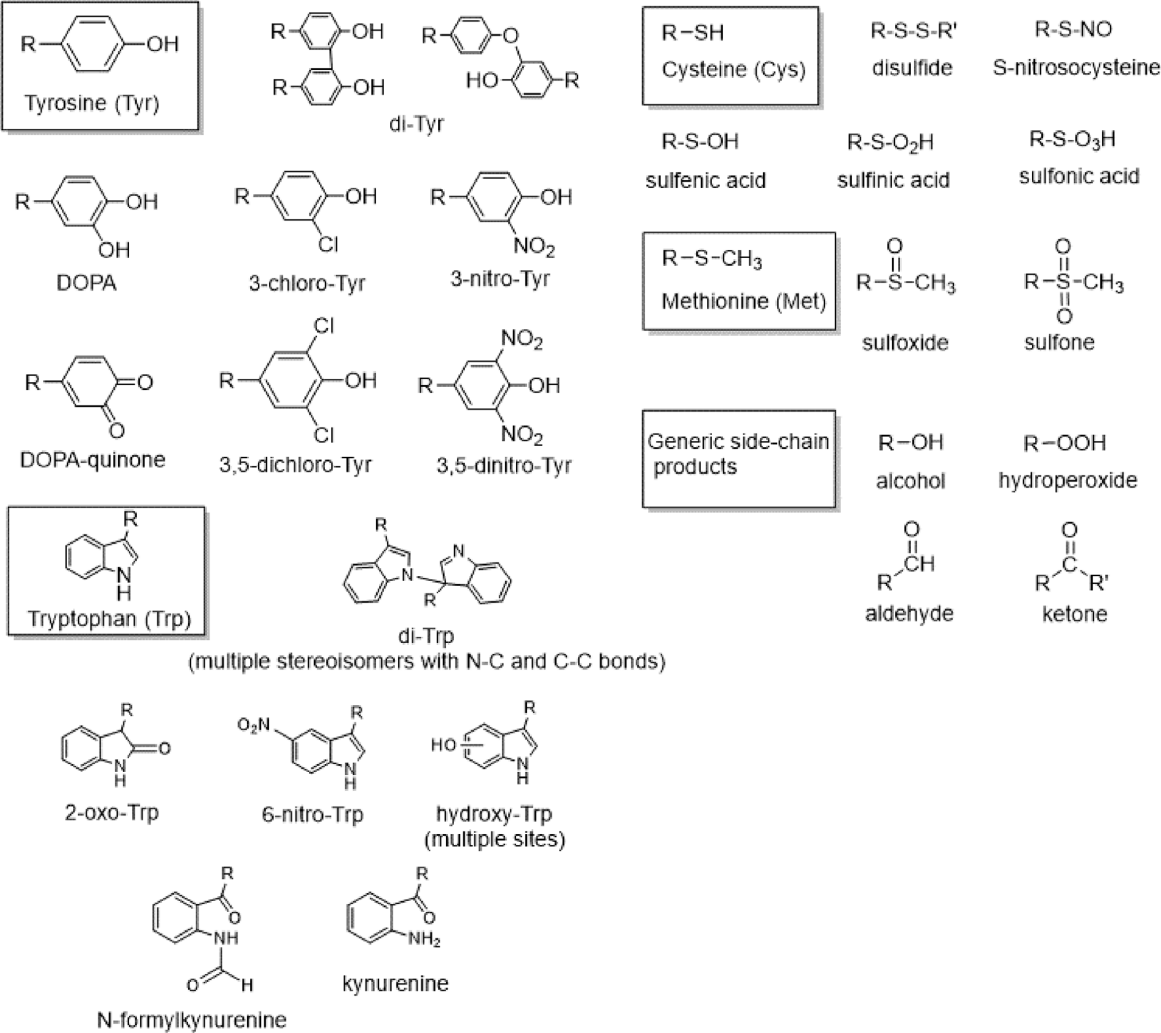
Chemical structure of the most common oxidation products detected on protein side-chains

As the chemical nature of protein modification induced by oxidants depend on the chemical properties of these, and on protein sequence, structure and surface dynamics, it is difficult to generalize or predict whether the damage will be reversible or irreversible, or the exact site of modification. Highly reactive oxidants, such as ^•^OH, indiscriminately oxidize common amino acids leading to the formation of a wide range of long-lived products (e.g. alcohols) and products that can either decompose or participate in secondary processes (e.g. hydroperoxides and carbonyls) [7,8]. Other one-electron and two-electron oxidants that despite being strong electrophiles are less reactive than ^•^OH (e.g. carbonate radical, CO_3_^•−^, peroxyl radicals, ROO^•^, singlet oxygen, ^1^O_2_), also lead to the formation of similar products. However, when these species are involved, oxidative modifications occur predominantly at aromatic and sulfur-containing side-chains (Figure 3). Additionally, oxidants such as hypohalous acids (e.g. hypochlorous acid, HOCl) and peroxynitrite (ONOO^−^) can lead to the formation of both general oxidation products and specific oxidation products such as 3-chloro-Tyr and 3-nitro-Tyr, respectively, that also impact protein structure and function (reviewed in [76–78]) (Figure 3). Furthermore, formation of secondary radical species at side-chains, or carbonyls, can induce protein intra- or intermolecular cross-linking. For example, radical-radical termination reactions between two Tyr phenoxyl radicals give di-Tyr crosslinks with *k* ∼ 10^9^ M^−1^ s^−1^ [79,80] (Figure 3). Similarly, tryptophanyl radicals generated after the abstraction of a hydrogen atom from the indole moiety of Trp residues can also participate in radical–radical termination reactions leading to di-Trp formation that induce protein dimerization [80,81]. Additionally, secondary reactions between (uncharacterized) carbonyl groups at His, Tyr, or Trp residues, or the Tyr oxidation product DOPA-quinone, with the nucleophilic residues Cys, Lys, or Arg (e.g. via Michael addition reactions) also lead to protein cross-linking [80,82,83]. The sulfur-containing amino acids Cys and Met can be oxidized to both reversible and non-reversible species, with the first type of products playing important roles for redox signaling in physiology (*vide supra*) and the second having profound impacts on protein structure, function and turnover. Cys overoxidation leads to the formation of the strong oxyacid products sulfinic (reversible only in the presence of specific reductases) and sulfonic acids (irreversible), which are negatively charged at physiological pH, and thus change the microenvironment where the residue sits. Met overoxidation results in the formation of the sulfone (MetSO_2_), which cannot be reverted.

## Macromolecular crowding as an important modulator of protein oxidation

**Figure 4:**
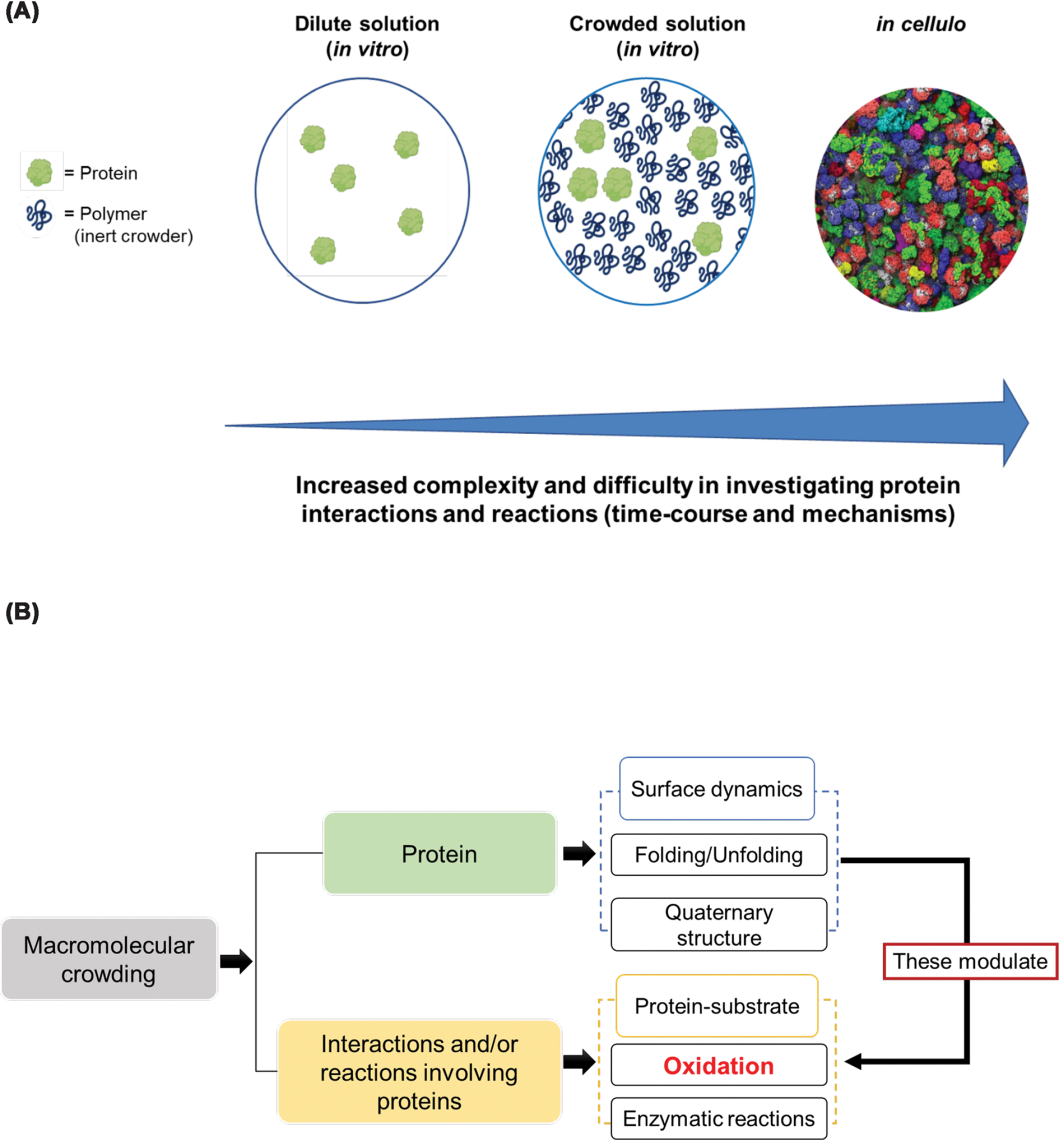
Schemes depicting the need to use crowded and heterogeneous systems to better understand reactions of biological relevance (**A**) Workflow to understand protein oxidation from a kinetic and mechanistic point of view in (crowded) biological environments. The *in cellulo* representation was reproduced under CC-BY-SA 3.0 from the cell image library. (**B**) Macromolecular crowding modulates protein biophysics (e.g. surface dynamics, folding, and protein–protein interactions), and also exerts an effect on biomolecular reactions. Thus, macromolecular crowding is an important feature to be considered when studying protein oxidation.

The oxidative modification of proteins depends on a number of factors including the physicochemical properties of the oxidant and the target residue. In addition, properties of the milieu where oxidation reactions take place, such as pH, and ionic strength (i.e. buffer composition), also stand as important modulators of the oxidative biology of proteins [84–86]. All these aspects have been historically considered in the experimental design. Nevertheless, there is another important feature of biological environments, that has a major impact on the physicochemical properties of the solvent, diffusion of molecules, and protein biophysics, has been less explored [13]. What I refer to is the fact that biological systems are commonly densely packed by a high amount of (macro)molecules that compose them. These densely packed conditions influence different aspects of protein biochemistry and biophysics, thus, may play an important role on protein oxidation reactions (Figure 4, panels A and B).

Molecular crowding is the term used to illustrate the highly occupied space within biological milieus, including the cell interior, as a result of the high concentration of the different molecules and macromolecules that compose these systems, with proteins standing as the most abundant macromolecules in both prokaryotes and eukaryotes [87]. This high abundance of (macro)molecules induces steric hindrance and serves as a driving force for the occurrence of non-specific interactions and macromolecular association events [87–89]. Furthermore, these crowded conditions also result in altered local viscosities due to hydrogen-bonding networks and electrostatic interactions generating dynamic “mosaic-like” regions that differ in composition and properties to the bulk system (i.e. spatial and compositional heterogeneity predominates in the cytoplasm) [90,91]. The latter may temporarily modulate the local concentration of proteins, substrates and/or other molecules including oxidants, thereby altering reaction rates and oxidation pathways [13]. A key conundrum is whether, and to what extent, biochemical reactions differ *in vivo* from results obtained *in vitro* as most experiments to address the effect of oxidative modifications on protein structure and function are typically conducted using dilute solutions.

The fundamental difference between processes studied *in vitro* versus the *in cellulo* situation lies on the excluded volume principle that arises from steric hindrance. This is a purely thermodynamic concept that establishes that the presence of “inert” macromolecules reduces the space available to target biomolecules. Consequently, any process that minimizes the volume excluded by the crowder, such as a transition to a more compact molecular state, becomes thermodynamically favored. The excluded volume effect is an important modulator of protein structure, folding, and conformational dynamics in biological systems [13,89]. For proteins existing in an equilibrium between an unfolded and a compact state, the introduction of inert crowder molecules (e.g. ficoll or dextran) consistently shifts the equilibrium toward the stabilization of the compact form [92–98]. This has been reported for the enzyme lysozyme, amongst other proteins [92,93,95,96]. Upon reductive denaturation of the enzyme, its yield of refolding is significantly favored toward its native conformation in the presence of mixed solutions containing bovine serum albumin, dextran and/or ficoll compared with dilute conditions [92]. However, this example cannot be generalized, as in a complex and heterogeneous milieu there must be also taken into account the critical kinetic competition where the compact, native fold is stabilized alongside non-native, compact aggregates. Thus, while crowding favors compact structures, it can also promote interactions between unfolded proteins enhancing other processes such as oligomerization and aggregation [99–101].

Additionally, crowded milieus are characterized by the altered local viscosities resulting in the formation of dynamic environments that behave closer to a gel than a solution exerting non-homogeneous effects on translational diffusion, association rates, and enzyme kinetics. The high degree of occupancy significantly alters the translational diffusion of target proteins and substrates, often leading to anomalous, non-Fickian diffusion, where the movement of molecules is sub-diffusive and characterized by temporary trapping within cages formed by the surrounding macromolecules. This high local viscosity and constrained mobility has a direct, profound impact on all diffusion-limited reactions (e.g. radical–radical termination reactions that lead to protein cross-linking, see above), reducing the collision frequency of target molecules compared with dilute solutions [13]. In contrast, the thermodynamic driving force of the excluded volume effect strongly favors those reactions that depend on the spatial arrangement of the reactants or depend on weak interactions that contribute to decreasing the activation energy of a given reaction. These processes are substantially favored under crowded conditions because the formation of the complex reduces the total molecular surface area, leading to an entropic gain from the release of displaced crowder molecules back into the bulk solution. This has been empirically confirmed for various systems, such as the enhanced binding affinity observed between proteins like Superoxide Dismutase and Xanthine Oxidase [102]. These effects on association events underscore that the cellular organization relying on transient protein interactions are substantially favored *in vivo*. Another example of this, is the prevalence of the tetrameric form of GAPDH over the monomeric or dimeric states, with the equilibria between these states being significantly shifted toward the oligomeric state under crowded conditions [103]. However, the kinetic effect on enzyme catalysis is highly specific and often non-uniform, proving that the chemical identity of the crowding agent and its potential for non-specific interactions must be considered alongside the excluded volume effect. While some enzymes, such as Adenylate Kinase, exhibit an overall enhancement in catalytic activity in the presence of crowders like ficoll, other enzymes display a significant decrease in their activity [104–107]. For example, the function of human Arginase-I significantly decreased in the presence of polyethylene glycol (PEG) [108]. This suppression was attributed to non-specific weak interactions between the PEG chains and the enzyme surface, which induce subtle, localized structural distortions within the catalytic pocket, thereby impeding optimal substrate access or the required dynamic motion for transition state formation. These observations imply that kinetic outcomes are highly dependent on the chemical properties, molecular mass and concentration of the crowders, emphasizing the necessary transition from viewing the cytoplasm as a simple uniform steric obstacle to a physicochemical effector where compositional heterogeneity is paramount.

Considering the above discussed, it is rational to sustain that the dense and heterogeneous environment generated by molecular crowding alters the oxidative modification of proteins, modulating the kinetic and thermodynamic landscape of damage from what has been reported in dilute models. Thus, physical hindrance, high local viscosities, altered diffusion of oxidants, and target proteins, together with the effects of crowding on protein conformation (including surface dynamics), have an important impact on the oxidative modification of proteins. A consequence of crowding on protein oxidation is its profound influence on free-radical chain reactions. As discussed in section 2, protein oxidation can result in the formation of secondary free radicals at side-chains ((eqn 1), where FR^•^= free radical), which, as depicted in (eqn 2), can subsequently react with molecular oxygen to form protein-derived peroxyl radicals, species able to further oxidize other side-chains propagating the oxidative damage (eqn 3).

(1) $$\text{Protein-H}+{\text{FR}}^{\text{•}}⟶{\text{Protein}}^{\text{•}}$$

(2) $${\text{Protein}}^{\text{•}}+{\text{O}}_{2}⟶{\text{Protein-OO}}^{\text{•}}$$

(3) $${\text{Protein-OO}}^{\text{•}}+\text{Protein-H}⟶\text{Protein-OOH}+{\text{Protein}}^{\text{•}}$$

(4) $${\text{Protein}}^{\text{•}}+{\text{Protein}}^{\text{•}}-\text{Dimers}$$

**Figure 5:**
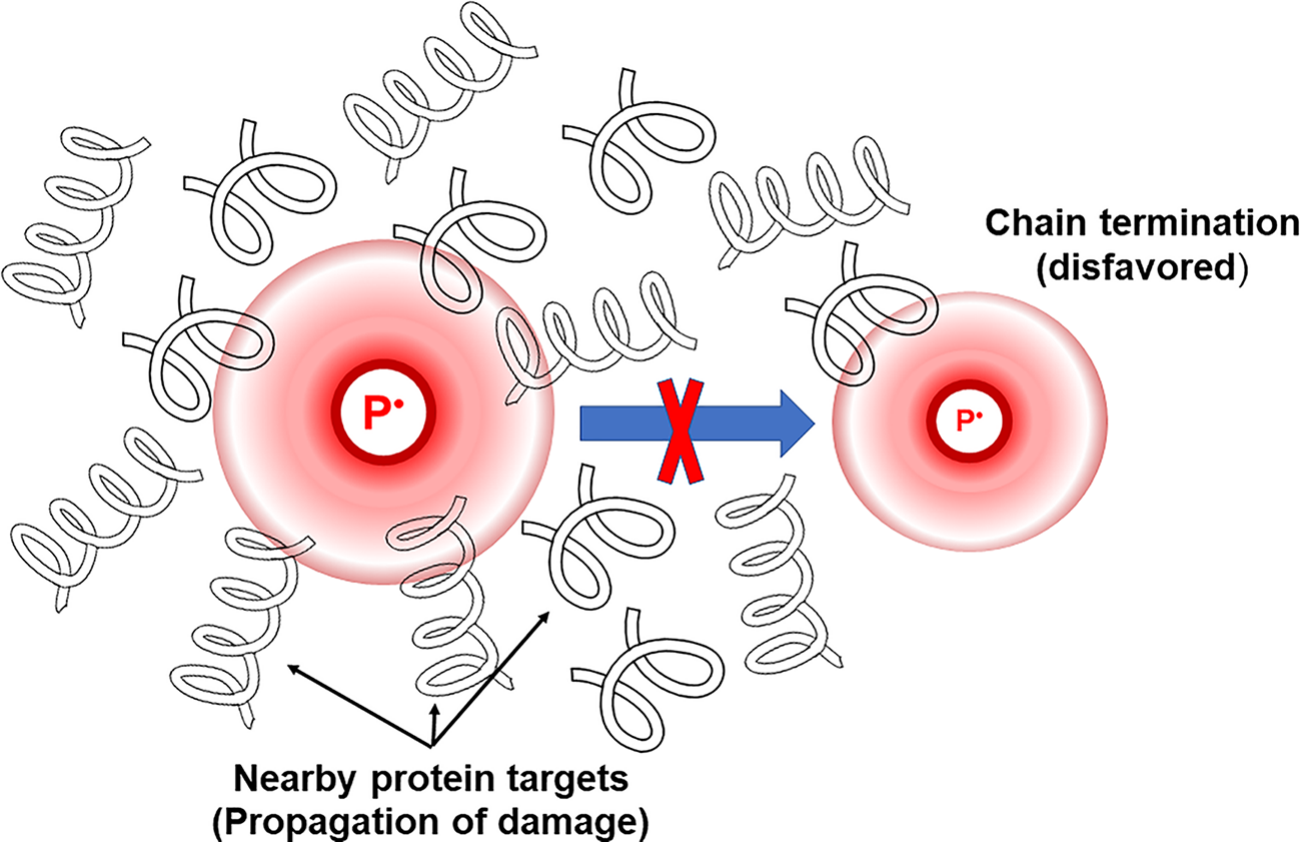
Macromolecular crowding affect protein oxidation pathways The recombination of a pair of protein-derived radical species (depicted as P^•^ in the scheme) is a diffusion-limited process. Thus, as crowding decreases the translational diffusion of P^•^ due to the increasing number of obstacles, the recombination of these species is disfavored. Contrarily, propagation of damage is favored as P^•^ is more likely to oxidize other proteins in the vicinity.

Under dilute conditions, these chain reactions that are initiated, propagated, and terminated by free radical species are often rapidly shut down by chain-terminating reactions (eqn 4), such as the combination of two short-lived, reactive free radicals to form stable products (e.g. recombination of two tyrosyl or tryptophanyl radicals to form di-Tyr or di-Trp) [80]. However, in biological systems, where proteins are packed together due to the crowded conditions, the translational diffusion of these secondary radical species is dramatically slowed down. This reduction in mobility leads to a kinetic decoupling of the chain propagation step (where a secondary radical attacks a protein side chain) from the chain termination step (where two radicals combine). Under these conditions, the secondary protein–radical species are more likely to react with a nearby, concentrated protein target than to diffuse quickly enough to find another radical for termination (Figure 5). This results in the propagation of damage and lengthening of the oxidation chains (similarly to lipid peroxidation processes [109]), meaning that a single initiating event causes a much greater number of subsequent oxidative modifications. One of the first reports rigorously describing the effect of concentrated protein solutions on the propagation of oxidative damage was conducted by Aicardo and co-workers (2018) [14]. The authors exposed bovine serum albumin at different concentrations (up to 4.5 mM, ∼300 mg ml^−1^) to peroxynitrite (ONOO^−^), or metmyoglobin/H_2_O_2_, to initiate the damage. Their results confirmed the formation of protein-centered thiyl radicals at Cys residues upon the reaction between BSA with the oxidants. These thiyl radicals were able to propagate damage through an oxygen-dependent radical mechanism in a concentration-dependent manner with a 3-fold longer propagation chain when BSA Cys-34 thiol group was reduced compared with when this residue was alkylated [14]. This observation (i.e. the effect of crowded solutions on the propagation of oxidative damage within proteins) was further confirmed by Fuentes-Lemus and co-workers (2021) [110]. In this study, the authors exposed the intrinsically disordered casein proteins to peroxyl radicals (ROO^•^), or to a photosensitized system (riboflavin/O_2_), leading to the formation of protein-centered radicals that in aerobic milieus form secondary peroxyl radicals (similarly to the process described in eqns 1 and 2). The results confirmed that the oxidative modification of casein proteins by the two different oxidation systems was highly dependent on the protein concentration, with protein cross-linking being the favored process at low protein concentrations, while at higher protein concentrations chain reactions predominated. Thus, the moles of amino acid residues oxidized on kappa casein per mole of ROO^•^ generated in the systems increased from ∼2.2 residues oxidized per mole of ROO^•^ generated to ∼10.5 residues oxidized per mole of ROO^•^ generated when the protein was incubated at a concentration of 1 and 27 mg ml^−1^, respectively. This study also confirmed the important role that Cys and Trp residues play on the propagation of the oxidative damage in concentrated protein solutions [110].

Beyond its effect on the extent of damage (via modulation of propagation reactions), macromolecular crowding also exerts a critical influence on oxidation pathways, particularly on Cys thiol oxidation pathways [16,111]. Cys residues are critical sensors for physiological oxidants and redox signaling (*vide supra*). In the context of reversible signaling, Cys residues in target proteins react with H_2_O_2_ to form the sulfenic acid (Cys-SOH) intermediate that can undergo further reversible or irreversible modifications [35]. Macromolecular crowding conditions introduce conformational alterations that can drive this reversible intermediate toward irreversible damage by promoting its hyper-oxidation to the sulfinic (Cys-SO_2_H) and sulfonic acid (Cys-SO_3_H) forms as observed for the glycolytic enzyme GAPDH [111]. This crucial shift in the oxidation pathways of signaling thiols in proteins may be mediated by two converging effects of molecular crowding. First, the high local viscosity and anomalous diffusion within the crowded milieu may significantly retard the diffusion of the antioxidant systems capable of reducing Cys-SOH, or other reversible species such as disulfides, back to Cys-SH in the active site of the signaling proteins. This restricted access would effectively “trap” the highly reactive Cys-SOH intermediate, giving it sufficient time to react with a second molecule of H_2_O_2_, thereby pushing it toward the irreversible hyper-oxidized products. Second, the combination of excluded volume effects and non-specific weak interactions induced by crowding can cause subtle, but functionally critical, conformational changes in the target protein. Studies on the key metabolic enzyme glyceraldehyde-3-phosphate dehydrogenase (GAPDH) have demonstrated that crowded conditions preferentially favor the formation of the irreversible Cys-SO_3_H form of its active site Cys upon reaction with high doses of H_2_O_2_ [111]. In this work, Glover and co-workers (2024) exposed GAPDH to a 40-fold molar excess of H_2_O_2_ under dilute and crowded conditions, and determined the relative levels of the oxyacids formed at the catalytic Cys residue by LC-MS [111]. Their results showed a decreased fraction of oxidized GAPDH, however, the analysis of the relative abundance of the oxyacids showed a significant increase in sulfonic acid formation from ∼17% (dilute condition) to ∼31% when incubation was carried out under crowded conditions. This suggests that the crowded environment may intrinsically increase the susceptibility of the catalytic Cys residue to hyper-oxidation, possibly by altering its local microenvironment, thereby decoupling the enzyme’s signaling capacity from its catalytic activity. Additionally, the presence of a high concentration of macromolecules may profoundly impact local viscosities altering the diffusivity of oxidants and co-factors themselves [13,91]. This would lead to the formation of nanodomains (*vide infra*) that differ in composition from the bulk environment, where the effective concentration of small-molecule substrates and oxidants, like H_2_O_2_, can be significantly higher near a reactive protein surface. This phenomenon may also contribute to synergistic modulation of oxidative pathways. This interplay has been observed upon exposure of GAPDH to H_2_O_2_ and bicarbonate (HCO_3_^−^) under crowded conditions [16]. In the crowded biological milieu, CO_2_ (in equilibrium with HCO_3_^−^) can react with H_2_O_2_ to form the highly reactive secondary oxidant peroxymonocarbonate (HCO_4_^−^) [15]. Although HCO_4_^−^ formation itself has been previously confirmed to enhance the oxidative modification of thiol-containing proteins under dilute conditions [28,112], incubation of GAPDH with H_2_O_2_ and HCO_3_^−^ under crowded conditions significantly enhanced the oxidative inactivation of the enzyme [16]. This synergistic effect underscored how the non-ideal physical chemistry of crowding may modulate the oxidative modification and inactivation of important signaling proteins.

Finally, the principles of crowded and heterogeneous solutions also govern the rates of other important post-translational modifications, such as glycation and glycoxidation [113,114]. Crowding conditions are observed to significantly enhance the efficiency of glycation of macromolecules like whey proteins, albumin, and transferrin. This acceleration is mechanistically attributed to the combined local concentration effects of crowding, which increase the effective frequency of collision between reactive α-oxoaldehydes and the nucleophilic side chains (e.g. Lys or the N-termini) on the protein surface, alongside potential conformational changes that expose reactive nucleophilic residues, leading to an amplified extent of molecular modification and subsequent structural alteration. This body of evidence collectively establishes macromolecular crowding not merely as a consequence of high cellular density but as an important feature that governs the structure, diffusion, interaction, and reaction of biological macromolecules highlighting the importance of using crowded non-heterogeneous systems to study protein oxidation and modification.

## Interplay between crowding, protein oxidation, and confinement in the biological context

The crowded intracellular environment is a key driver for the liquid–liquid phase separation (LLPS) of biomolecules (i.e. confinement). As previously discussed, this important feature of biological systems (i.e. crowding) not only generates steric hindrance, it also favors non-specific interactions. Thus, it favors the confinement of biological macromolecules based both on attractive chemical interactions between the phase-separating components, and also through the ubiquitous, non-specific and entropically favored phenomenon known as depletion attraction [115,116]. Inside the cell, where the occupied volume varies between 30% and 40%, the system seeks to maximize its overall free energy by optimizing the location and configuration of its components. This optimization is achieved by reducing the total excluded volume, thereby maximizing the translational entropy of the crowding molecules. The depletion force arises when two or more phase-separating macromolecules (i.e. the components destined for the dense phase, such as proteins), get in close proximity within the highly occupied local milieu. When the distance between these macromolecules becomes smaller than the diameter of other “inert” molecules in the vicinity, the volume that the crowding agent could previously access is lost. To recover this inaccessible volume and increase their available configurations (i.e. maximize their entropy), the bulk solution of crowding agents exerts a net force on the molecules that are destined for the dense phase. This force effectively pushes the phase-separating components together. This gain in the configurational entropy of the crowding species provides a powerful, non-specific attractive potential, which dramatically stabilizes the condensed phase and establishes crowding as the necessary prerequisite for phase separation under physiological conditions. The immediate consequence of this depletion effect is the shift in the phase boundary of LLPS. For any given protein capable of undergoing phase separation, the crowded environment decreases the critical concentration at which this happens compared with a dilute solution [117–120]. This means that proteins behave completely differently in a test tube (under dilute conditions) compared with the thermodynamically favored state in the crowded cell. Thus, biomolecular condensates form rapidly and transiently in response to small, localized stimuli within the cell. The latter has been confirmed by several studies employing model crowding agents like PEG, ficoll, and dextran, demonstrating that crowding is essential for LLPS, affecting not just the phase boundary but also the resulting condensate properties [117–124].

Yet, while macromolecular crowding provides the essential thermodynamic driving force for LLPS, the cell requires an additional, highly localized and rapid mechanism to control the formation and dissociation of biomolecular condensates. This dynamic control has been linked to localized changes in the concentration of ions (e.g. Ca^2+^), ATP, or in response to other important metabolites such as citrate [125–131]. In addition, reversible oxidative post-translational modifications (i.e. protein oxidation) are also recognized as important contributors to condensate formation [132–137]. The chemical modification of protein sidechains driven by an oxidative signal provides the final, targeted shift needed to form stable and reversible condensates. The reversible oxidation of proteins by locally formed oxidants such as H_2_O_2_ or other important biological oxidants (*vide supra*) induces structural modifications on specific Cys or Met residues that can either favor or disfavor protein–protein interactions, adding a new force that stabilize or destabilize condensates. This has been observed for different proteins including Vimentin, GAPDH and the DNA-binding protein hSSB1, amongst other proteins [132,133,135,137,138]. Vimentin is a stable and highly structured type III intermediate filament protein that alongside other filament proteins form the cytoskeleton. However, oxidation of specific Cys residues upon exposure to oxidants (e.g. H_2_O_2_ or diamide) induces conformational changes that disrupt the interactions needed for filament assembly remodeling the protein and shifting the energetic balance to favor other weaker interactions that result in LLPS [135]. This process triggers a switch from stable filamentous networks to dynamic, phase-separated condensates. In the crowded cytosol, these reversible oxidative modifications are hypothesized to induce the necessary conformation changes to overcome the energy barrier to form the stress-response condensates. Another example of a protein undergoing redox-sensitive LLPS is the glycolytic enzyme GAPDH. This moonlighting enzyme plays critical roles including its main function as a central metabolic enzyme in the cytoplasm and a secondary function as a nuclear signaling protein under stress conditions [139]. This shift in function is directly regulated by conformational state and oxidation [140,141]. Increasing levels of H_2_O_2_ induce the oxidation of the reactive catalytic Cys152 residue in GAPDH, leading to the inactivation of its glycolytic function. This chemical modification acts as a structural allosteric trigger, causing a conformational change that favors the formation of multivalent intermolecular interactions [138]. These altered interactions promote the sequestration of GAPDH into cytoplasmic stress granules, a type of biomolecular condensate. As described by Vignane and co-workers (2023), this phase-separation event effectively serves two purposes, it temporarily shuts down non-essential glycolytic activity to conserve energy, and it allows the oxidized form of GAPDH to participate in stress–response pathways, demonstrating a direct mechanistic link where the oxidation of a specific, critical residue converts a metabolic enzyme into a phase-separating signaling node [138]. Conversely, exposure of the Splicing Factor Proline/Glutamine Rich protein (SFPQ, an RNA-binding protein) to H_2_O_2_, showed that the redox state of the SFPQ is critical for condensate formation, with data showing an H_2_O_2_-dose dependent dissolution of condensates [142]. Thus, when SFPQ was oxidized at critical Cys or Met residues within the protein structure, SFPQ condensates were dissolved favoring formation of stress granules that would play a role in Amyotrophic Lateral Sclerosis [142,143]. A similar observation has been reported for yeast proteins, where protein oxidation has been shown to disrupt the weak interactions that allow LLPS [132]. The Ataxin-2 homolog protein (Pbp1), undergoes condensate formation where its Met-rich low-complexity domain (LCD) is key. Under distress conditions, H_2_O_2_ triggers the oxidation of these Met residues to MetSO. Oxidation of Met residues in the LCD region changes its polarity disrupting the weak interactions that are essential for stabilizing the condensed polymer, leading to polymer dissolution and the release of Pbp1 back into the soluble phase. This mechanism serves as a rapid safety switch that can be reversed by MetSO reductases.

**Figure 6:**
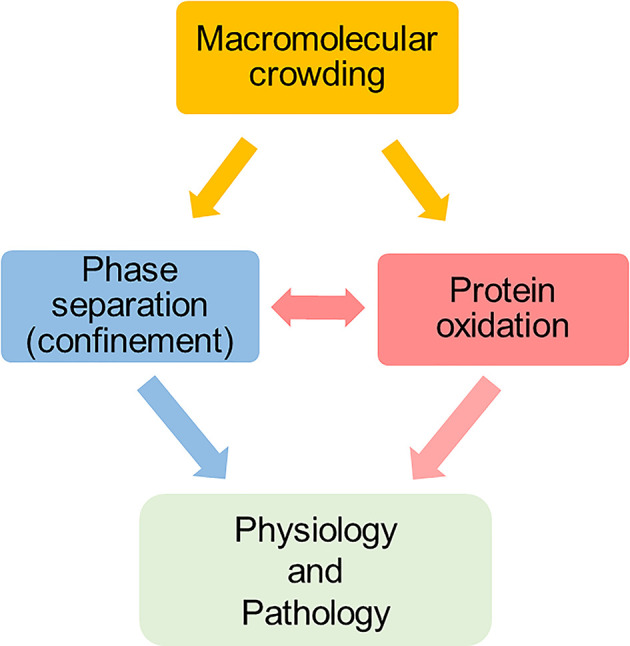
Macromolecular crowing is a driving force for phase separation and modulates protein oxidation Macromolecular crowing is a driving force for phase separation (i.e. protein confinement) and modulates protein oxidation. Additionally, there is an important interplay between protein confinement and protein oxidation, with these playing important roles in physiology and pathology.

These examples illustrate the interplay between protein oxidation and condensate formation, with these local events taking place within the heavily crowded milieu (Figure 6). Interestingly, the interplay between crowding, protein oxidation and condensate formation is not only relevant under physiological conditions to explain cell adaptation. These are also relevant in the context of the molecular basis of pathology. If oxidative modifications shift from formation of reversible products to irreversible species, then the highly dynamic nature of condensates is lost. General protein thiol alterations resulting from persistent oxidative stress have been directly linked to the formation of aberrant phase separation associated with aging and neurodegenerative diseases [144]. In this context, the initial entropic drive provided by crowding is no longer utilized for reversible organization but leads to irreversible aggregation, where the liquid-like state transitions into a solid-like, pathological structure. The inability to reverse the oxidative modifications, or the formation of irreversible cross-links, may lock the confined proteins into a non-functional state.

## Methodological strategies to study protein oxidation in crowded systems

The study of protein oxidation in crowded milieus requires the adaptation of classical biochemical and biophysical techniques. However, multiple factors need to be considered to mimic the crowded, complex, and dynamic heterogeneities found in biological milieus. Within the cell, crowding is not generated by a single type of molecule or macromolecule; instead, there is a wide range of species of markedly different size, conformation and chemical properties (e.g. presence of heteroatoms, and charged versus uncharged molecules). Therefore, to successfully address the effect of crowding on protein oxidation, and the signals these modifications relay, there is a need to combine quantitative and mechanistic *in vitro* assays that utilize well-defined crowding agents with spatially resolved *in cellulo* techniques [13,145]. Additionally, high-resolution *in silico* modeling also arises as a powerful approach to study the structure-function–environment relationship.

For *in vitro* studies aiming to investigate protein oxidation pathways and kinetics, the selection of the crowding agents to be utilized is not trivial as the interpretation of the results will depend on these. Inert polymers, such as ficoll or dextran, offer the advantage of well-known physico-chemical properties and generation of the excluded volume phenomenon [146,147]. These inert polymers favor compact protein structures, alter diffusion and generate dynamic domains. Thus, these are a good alternative to study the effects of these aspects of biological systems on the oxidative modification of proteins. Furthermore, their chemical composition makes these inert to most biological oxidants with exception of those highly reactive (e.g. ^•^OH) minimizing competition reactions that will result in difficult result interpretation. However, their disadvantage lies in their lack of physiological realism as they do not account for the heterogeneous surface interactions and weak attractive/repulsive forces present in the cell. However, there exist modified polymers with functional groups that can present negative or positive charges adding more complexity to the system, but not all these polymers are suitable for spectroscopic studies and batch-to-batch purity should be considered for reproducibility. Our group has recently reported that dextran and ficoll can be used to obtain kinetic data on protein oxidation, and to investigate the oxidative inactivation of important metabolic and signaling proteins by oxidants of modest oxidation potential, and also for glycation agents [113,148]. Furthermore, existing protocols to rigorously investigate general and specific protein oxidation makers can be adapted to from dilute studies to crowded studies as long as control experiments with the crowding agents are previously performed and confirmed not to interact with reagents used to derivatize common oxidation markers such as carbonyls and hydroperoxides [149,150]. In contrast, using a high-concentration protein mixture (e.g. total cell lysate or abundant non-signaling proteins) as the crowder has the advantage of better mimicking the complex chemical and physical heterogeneity of the cytoplasm. Nevertheless, the disadvantage of this strategy lies in the difficulty in deconvoluting the effect of the excluded volume effect from specific non-covalent interactions, together with the competition that will exist between the target protein under study from the high background concentration of other proteins that may also be targeted by the oxidant molecule(s).

Additionally, both *in vitro* and *in cellulo* approaches to study protein oxidation in crowded milieus benefit from the high analytical resolution achieved by new mass spectrometer machines. Proteomic approaches (bottom-up and top-down) are ideal to characterize oxidative modifications and map the modified sites to achieve a greater understanding of the structure-modification-consequence relationship. For example, by comparing the ratio of Cys-SO_3_H and Cys-SOH formation in dilute versus crowded systems, it is possible to precisely quantify the crowding-induced shift in the oxidative fate of the oxidation of key Cys residues, thereby providing direct evidence for the mechanistic impact of excluded volume on chemical reactions [111]. Finally, *in cellulo* studies must overcome the focus of studying bulk oxidation processes. This can be addressed by shifting from global measurements (e.g. total protein hydroperoxides or total protein carbonyls) to spatially resolved redox sensing [151–153]. As reported by Kritsiligkou (2023), the adaptation of genetically encoded biosensors for H_2_O_2_ to a proteome-wide tagging strategy allows to identify highly localized and dynamic redox microenvironments that are generated by the restricted diffusion and organization of macromolecules inherent to the crowded environment [153]. This is a crucial adaptation for understanding the interplay between protein oxidation and redox signaling, as it links the physical constraints of crowding and compartmentalization to the generation and transmission of specific oxidative signals *in vivo*. These empirical measurements of diffusion and localization can serve as the necessary input parameters for computational approaches [154]. Then, these can be adapted to simulate reaction kinetics under the diffusion-limited constraints imposed by crowding, providing the mechanistic link between the physical environment and the oxidation processes.

## Concluding remarks and perspectives

The literature discussed in this invited review article aimed to illuminate the effect of crowded milieus on protein oxidation and confinement. The redox biochemistry field is moving beyond viewing these phenomena as independent variables currently recognizing them as integrated regulatory controls that define cell physiology and that can also contribute to pathology. While complex concepts such as the excluded volume phenomenon that arises from crowded milieus, and the central role of reversible protein oxidation for redox signaling, are well-established and count with several studies supporting these with robust mechanistic, kinetic and structural data, the future of this field lies in addressing the complexity and heterogeneity inherent of living systems. Thus, I foresee the need to rigorously address three critical areas to overcome our current knowledge limitations. The first one, is to continue elucidating protein oxidation pathways under crowded conditions, recognizing this as the core challenge for understanding redox signal specificity. Hence, future works aimed to illuminate the effect of oxidative modifications on protein structure and function from a mechanistic or kinetic point of view should consider the effect of macromolecular crowding as an important modulator of the redox biology of proteins. This emphasis on crowding is of major relevance because the cellular environment is not simply an inert dilute buffer, but an active modulator of oxidative damage. For example, there exist critical redox sensor proteins such as peroxiredoxins (Prdxs) that exist in a dynamic equilibrium between different oligomeric and redox states [155]. Particularly, eukaryotic 2-Cys Prdxs depend on local rearrangements to form a disulfide between the peroxidatic Cys and the resolving Cys. If formation of the disulfide is structurally impeded, this may favor the hyperoxidation of the peroxidatic Cys affecting the reversibility of the modification. This highlights the importance of establishing the structure–function relationship of important redox sensing proteins under conditions mimicking the crowded intracellular milieu.

Secondly, the field must move beyond bulk redox changes to map specific, transient H_2_O_2_ “hotspots” and redox nanodomains occurring at the level of individual protein complexes and phase-separated compartments, for which the use of advanced biosensors is mandatory. This will contribute to continue deciphering the role of the crowded intracellular milieu on redox processes and establish these as important phase switches that regulate signaling pathways. In particular, the formation and dissociation of biomolecular condensates (LLPS) must be detected and quantified to confirm that the redox state and the degree of crowding function as direct regulators of these phase transitions. Specifically, a primary research goal would be defining the molecular mechanisms by which protein thiol oxidation drives aberrant phase separation and the subsequent formation of pathological aggregates, hallmarks of aging and neurodegenerative diseases.

The final key direction is identifying the specific oxidative modifications that relay information to signaling pathways in such complex and heterogeneous milieus, that end up driving adaptive changes essential for physicochemical and biochemical homeostasis in all cell types. To successfully navigate these future directions, the field requires a significant shift in experimental approaches, moving away from simplified dilute-solution biochemistry toward complex, physiologically relevant systems. The experimental roadmap must include continuing developing high-resolution biosensors alongside the use of proteomics to identify, track and characterize dynamic oxidation events *in vivo*. Furthermore, *in vitro* studies must be conducted under conditions that mimic the biological situation by adding heterogeneous crowding agents and relevant co-solutes. In conclusion, the convergence of protein oxidation, redox signaling, macromolecular crowding, and phase separation represents an important frontier of cell biology, and only by embracing new (bio)chemical tools and rigorous experimental designs that respect the crowded and heterogeneous nature of the cell, we will unlock fundamental regulatory principles critical for understanding cellular homeostasis, aging, and a wide spectrum of human pathologies.

## Data Availability

This review manuscript does not contain original research data, thus, should any raw data files of the discussed works be needed, these must be requested to the corresponding author(s) of the referenced documents.

## Abbreviations

LCD: low-complexity domain
LLPS: liquid–liquid phase separation
PEG: polyethylene glycol
SFPQ: Splicing Factor Proline/Glutamine Rich

## References

[1] Sies H. and Jones D.P. (2020) Reactive oxygen species (ROS) as pleiotropic physiological signalling agents. Nat. Rev. Mol. Cell Biol. 21, 363–383 10.1038/s41580-020-0230-3 32231263

[2] Sies H., Belousov V.V., Chandel N.S., Davies M.J., Jones D.P., Mann G.E. et al. (2022) Defining roles of specific reactive oxygen species (ROS) in cell biology and physiology. Nat. Rev. Mol. Cell Biol. 23, 499–515 10.1038/s41580-022-00456-z 35190722

[3] Sies H., Mailloux R.J. and Jakob U. (2024) Fundamentals of redox regulation in biology. Nat. Rev. Mol. Cell Biol. 25, 701–719 10.1038/s41580-024-00730-2 38689066 PMC11921270

[4] Cooke M.S., Evans M.D., Dizdaroglu M. and Lunec J. (2003) Oxidative DNA damage: mechanisms, mutation, and disease. FASEB J. 17, 1195–1214 10.1096/fj.02-0752rev 12832285

[5] Sousa B.C., Pitt A.R. and Spickett C.M. (2017) Chemistry and analysis of HNE and other prominent carbonyl-containing lipid oxidation compounds. Free Radic. Biol. Med. 111, 294–308 10.1016/j.freeradbiomed.2017.02.003 28192230

[6] Reis A. and Spickett C.M. (2012) Chemistry of phospholipid oxidation. Biochim. Biophys. Acta - Biomembr. 1818, 2374–2387 10.1016/j.bbamem.2012.02.002 22342938

[7] Davies M.J. (2005) The oxidative environment and protein damage. Biochim. Biophys. Acta - Proteins Proteomics 1703, 93–109 10.1016/j.bbapap.2004.08.007 15680218

[8] Davies M.J. (2016) Protein oxidation and peroxidation. Biochem. J. 473, 805–825 10.1042/BJ20151227 27026395 PMC4819570

[9] Kehm R., Baldensperger T., Raupbach J. and Höhn A. (2021) Protein oxidation – formation mechanisms, detection and relevance as biomarkers in human diseases. Redox Biol. 42, 101901 10.1016/j.redox.2021.101901 33744200 PMC8113053

[10] Milo R. (2013) What is the total number of protein molecules per cell volume? A call to rethink some published values Bioessays 35, 1050–1055 10.1002/bies.201300066 24114984 PMC3910158

[11] Xiao H., Jedrychowski M.P., Schweppe D.K., Huttlin E.L., Yu Q., Heppner D.E. et al. (2020) A quantitative tissue-specific landscape of protein redox regulation during aging. Cell 180, 968.e24–983.e24 10.1016/j.cell.2020.02.012 32109415 PMC8164166

[12] Aebersold R., Agar J.N., Amster I.J., Baker M.S., Bertozzi C.R., Boja E.S. et al. (2018) How many human proteoforms are there? Nat. Chem. Biol. 14, 206–214 10.1038/nchembio.2576 29443976 PMC5837046

[13] Fuentes-Lemus E. and Davies M.J. (2023) Effect of crowding, compartmentalization and nanodomains on protein modification and redox signaling – current state and future challenges. Free Radic. Biol. Med. 196, 81–92 10.1016/j.freeradbiomed.2023.01.011 36657730

[14] Aicardo A., Mastrogiovanni M., Cassina A. and Radi R. (2018) Propagation of free-radical reactions in concentrated protein solutions. Free Radic. Res. 52, 159–170 10.1080/10715762.2017.1420905 29278949

[15] Radi R. (2022) Interplay of carbon dioxide and peroxide metabolism in mammalian cells. J. Biol. Chem. 298, 102358 10.1016/j.jbc.2022.102358 35961463 PMC9485056

[16] Bloemen R.H.J., Radi R., Davies M.J. and Fuentes-Lemus E. (2024) Macromolecular crowding and bicarbonate enhance the hydrogen peroxide-induced inactivation of glyceraldehyde-3-phosphate dehydrogenase. Biochem. J. 481, 1855–1866 10.1042/BCJ20240597 39556220 PMC11668361

[17] Winterbourn C.C. and Hampton M.B. (2008) Thiol chemistry and specificity in redox signaling. Free Radic. Biol. Med. 45, 549–561 10.1016/j.freeradbiomed.2008.05.004 18544350

[18] Richardson D.E., Regino C.A., Yao H. and Johnson J.V. (2003) Methionine oxidation by peroxymonocarbonate, a reactive oxygen species formed from CO_2_/bicarbonate and hydrogen peroxide. Free Radic. Biol. Med. 35, 1538–1550 10.1016/j.freeradbiomed.2003.08.019 14680677

[19] Hawkins C.L. and Davies M.J. (2001) Generation and propagation of radical reactions on proteins. Biochim. Biophys. Acta - Bioenerg. 1504, 196–219 10.1016/S0005-2728(00)00252-8 11245785

[20] Neužil J., Gebicki J.M. and Stocker R. (1993) Radical-induced chain oxidation of proteins and its inhibition by chain-breaking antioxidants. Biochem. J. 293, 601–606 10.1042/bj2930601 8352726 PMC1134408

[21] Schöneich C. (2020) Radical rearrangement and transfer reactions in proteins. Essays Biochem. 64, 87–96 10.1042/EBC20190046 31922197

[22] Perluigi M., Di Domenico F. and Butterfield D.A. (2024) Oxidative damage in neurodegeneration: roles in the pathogenesis and progression of Alzheimer disease. Physiol. Rev. 104, 103–197 10.1152/physrev.00030.2022 37843394 PMC11281823

[23] An X., Yu W., Liu J., Tang D., Yang L. and Chen X. (2024) Oxidative cell death in cancer: mechanisms and therapeutic opportunities. Cell Death Dis. 15, 556 10.1038/s41419-024-06939-5 39090114 PMC11294602

[24] Piacenza L., Zeida A., Trujillo M. and Radi R. (2022) The superoxide radical switch in the biology of nitric oxide and peroxynitrite. Physiol. Rev. 102, 1881–1906 10.1152/physrev.00005.2022 35605280

[25] Dean R.T., Fu S., Stocker R. and Davies M.J. (1997) Biochemistry and pathology of radical-mediated protein oxidation. Biochem. J. 324, 1–18 10.1042/bj3240001 9164834 PMC1218394

[26] Trujillo M., Alvarez B. and Radi R. (2016) One- and two-electron oxidation of thiols: mechanisms, kinetics and biological fates. Free Radic. Res. 50, 150–171 10.3109/10715762.2015.1089988 26329537

[27] Carballal S., Radi R., Kirk M.C., Barnes S., Freeman B.A. and Alvarez B. (2003) Sulfenic acid formation in human serum albumin by hydrogen peroxide and peroxynitrite. Biochemistry 42, 9906–9914 10.1021/bi027434m 12924939

[28] Winterbourn C.C., Peskin A.V., Kleffmann T., Radi R. and Pace P.E. (2023) Carbon dioxide/bicarbonate is required for sensitive inactivation of mammalian glyceraldehyde-3-phosphate dehydrogenase by hydrogen peroxide. Proc. Natl. Acad. Sci. U.S.A. 120, e2221047120 10.1073/pnas.2221047120 37098065 PMC10161126

[29] Denu J.M. and Tanner K.G. (1998) Specific and reversible inactivation of protein tyrosine phosphatases by hydrogen peroxide: evidence for a sulfenic acid intermediate and implications for redox regulation. Biochemistry 37, 5633–5642 10.1021/bi973035t 9548949

[30] Portillo-Ledesma S., Randall L.M., Parsonage D., Dalla Rizza J., Karplus P.A., Poole L.B. et al. (2018) Differential kinetics of two-cysteine peroxiredoxin disulfide formation reveal a novel model for peroxide sensing. Biochemistry 57, 3416–3424 10.1021/acs.biochem.8b00188 29553725 PMC6383210

[31] Winterbourn C.C. (2008) Reconciling the chemistry and biology of reactive oxygen species. Nat. Chem. Biol. 4, 278–286 10.1038/nchembio.85 18421291

[32] Forman H.J., Maiorino M. and Ursini F. (2010) Signaling functions of reactive oxygen species. Biochemistry 49, 835–842 10.1021/bi9020378 20050630 PMC4226395

[33] Murphy M.P., Holmgren A., Larsson N.-G., Halliwell B., Chang C.J., Kalyanaraman B. et al. (2011) Unraveling the biological roles of reactive oxygen species. Cell Metab. 13, 361–366 10.1016/j.cmet.2011.03.010 21459321 PMC4445605

[34] Sies H. (2017) Hydrogen peroxide as a central redox signaling molecule in physiological oxidative stress: oxidative eustress. Redox Biol. 11, 613–619 10.1016/j.redox.2016.12.035 28110218 PMC5256672

[35] Paulsen C.E. and Carroll K.S. (2013) Cysteine-mediated redox signaling: chemistry, biology, and tools for discovery. Chem. Rev. 113, 4633–4679 10.1021/cr300163e 23514336 PMC4303468

[36] Hanschmann E.-M., Godoy J.R., Berndt C., Hudemann C. and Lillig C.H. (2013) Thioredoxins, glutaredoxins, and peroxiredoxins—molecular mechanisms and health significance: from cofactors to antioxidants to redox signaling. Antioxid. Redox Signal. 19, 1539–1605 10.1089/ars.2012.4599 23397885 PMC3797455

[37] Meyer Y., Buchanan B.B., Vignols F. and Reichheld J.-P. (2009) Thioredoxins and glutaredoxins: unifying elements in redox biology. Annu. Rev. Genet. 43, 335–367 10.1146/annurev-genet-102108-134201 19691428

[38] Lee S., Kim S.M. and Lee R.T. (2013) Thioredoxin and thioredoxin target proteins: from molecular mechanisms to functional significance. Antioxid. Redox Signal. 18, 1165–1207 10.1089/ars.2011.4322 22607099 PMC3579385

[39] Jacob C., Holme A.L. and Fry F.H. (2004) The sulfinic acid switch in proteins. Org. Biomol. Chem. 2, 1953–1956 10.1039/B406180B 15254616

[40] Akter S., Fu L., Jung Y., Conte M.L., Lawson J.R., Lowther W.T. et al. (2018) Chemical proteomics reveals new targets of cysteine sulfinic acid reductase. Nat. Chem. Biol. 14, 995–1004 10.1038/s41589-018-0116-2 30177848 PMC6192846

[41] Woo H.A., Jeong W., Chang T.-S., Park K.J., Park S.J., Yang J.S. et al. (2005) Reduction of cysteine sulfinic acid by sulfiredoxin is specific to 2-Cys peroxiredoxins. J. Biol. Chem. 280, 3125–3128 10.1074/jbc.C400496200 15590625

[42] Nelson K.J., Bolduc J.A., Wu H., Collins J.A., Burke E.A., Reisz J.A. et al. (2018) H_2_O_2_ oxidation of cysteine residues in c-Jun N-terminal kinase 2 (JNK2) contributes to redox regulation in human articular chondrocytes. J. Biol. Chem. 293, 16376–16389 10.1074/jbc.RA118.004613 30190325 PMC6200941

[43] Lee K. and Esselman W.J. (2002) Inhibition of PTPs by H_2_O_2_ regulates the activation of distinct MAPK pathways. Free Radic. Biol. Med. 33, 1121–1132 10.1016/S0891-5849(02)01000-6 12374624

[44] Hurd T.R., Collins Y., Abakumova I., Chouchani E.T., Baranowski B., Fearnley I.M. et al. (2012) Inactivation of pyruvate dehydrogenase kinase 2 by mitochondrial reactive oxygen species. J. Biol. Chem. 287, 35153–35160 10.1074/jbc.M112.400002 22910903 PMC3471752

[45] Paulsen C.E. and Carroll K.S. (2010) Orchestrating redox signaling networks through regulatory cysteine switches. ACS Chem. Biol. 5, 47–62 10.1021/cb900258z 19957967 PMC4537063

[46] Hildebrandt T., Knuesting J., Berndt C., Morgan B. and Scheibe R. (2015) Cytosolic thiol switches regulating basic cellular functions: GAPDH as an information hub? Biol. Chem. 396, 523–537 10.1515/hsz-2014-0295 25581756

[47] Winterbourn C.C. (2025) Peroxiredoxins: antioxidant activity, redox relays, and redox signaling. Biochemistry 64, 4477–4486 10.1021/acs.biochem.5c00500 41201371

[48] Anastasiou D., Poulogiannis G., Asara J.M., Boxer M.B., Jiang J., Shen M. et al. (2011) Inhibition of pyruvate kinase M2 by reactive oxygen species contributes to cellular antioxidant responses. Science 334, 1278–1283 10.1126/science.1211485 22052977 PMC3471535

[49] Schöneich C. (2005) Methionine oxidation by reactive oxygen species: reaction mechanisms and relevance to Alzheimer’s disease. Biochim. Biophys. Acta - Proteins Proteomics 1703, 111–119 10.1016/j.bbapap.2004.09.009 15680219

[50] Davies M.J. (2025) Methionine oxidation products as biomarkers of oxidative damage to proteins and modulators of cellular metabolism and toxicity. Redox Biochem. Chem. 12, 100052 10.1016/j.rbc.2025.100052

[51] Boschi-Muller S., Olry A., Antoine M. and Branlant G. (2005) The enzymology and biochemistry of methionine sulfoxide reductases. Biochim. Biophys. Acta - Proteins Proteomics 1703, 231–238 10.1016/j.bbapap.2004.09.016 15680231

[52] Miernyk J.A., Johnston M.L., Huber S.C., Tovar-Méndez A., Hoyos E. and Randall D.D. (2009) Oxidation of an adjacent methionine residue inhibits regulatory seryl-phosphorylation of pyruvate dehydrogenase. Proteomics Insights 2, PRI.S2799 10.4137/PRI.S279920622924

[53] Oien D.B., Carrasco G.A. and Moskovitz J. (2011) Decreased phosphorylation and increased methionine oxidation of α-Synuclein in the methionine sulfoxide reductase A knockout mouse. J. Amino Acids 2011, 1–6 10.4061/2011/721094 PMC3275937 22332004

[54] Kanayama A., Inoue J., Sugita-Konishi Y., Shimizu M. and Miyamoto Y. (2002) Oxidation of IκBα at methionine 45 is one cause of taurine chloramine-induced Inhibition of NF-κB activation. J. Biol. Chem. 277, 24049–24056 10.1074/jbc.M110832200 11983684

[55] Moskovitz J. and Smith A. (2021) Methionine sulfoxide and the methionine sulfoxide reductase system as modulators of signal transduction pathways: a review. Amino Acids 53, 1011–1020 10.1007/s00726-021-03020-9 34145481

[56] Reyes J.S., Fuentes-Lemus E., Fierro A., Rivero-Rodríguez K., Arenas F., Davies M.J. et al. (2025) Inactivation of human glucose 6-phosphate dehydrogenase (G6PDH) by peroxyl radicals is strongly modulated by its substrate and cofactor. Free Radic. Biol. Med. 233, 55–69 10.1016/j.freeradbiomed.2025.03.030 40120653

[57] Fucci L., Oliver C.N., Coon M.J. and Stadtman E.R. (1983) Inactivation of key metabolic enzymes by mixed-function oxidation reactions: Possible implication in protein turnover and ageing. Proc. Natl. Acad. Sci. U.S.A. 80, 1521–1525 10.1073/pnas.80.6.1521 6572914 PMC393633

[58] Chen C.-H., Li W., Sultana R., You M.-H., Kondo A., Shahpasand K. et al. (2015) Pin1 cysteine-113 oxidation inhibits its catalytic activity and cellular function in Alzheimer’s disease. Neurobiol. Dis. 76, 13–23 10.1016/j.nbd.2014.12.027 25576397 PMC4423621

[59] Dóka É., Ida T., Dagnell M., Abiko Y., Luong N.C., Balog N. et al. (2020) Control of protein function through oxidation and reduction of persulfidated states. Sci. Adv. 6, 10.1126/sciadv.aax8358 31911946 PMC6938701

[60] Wolff S.P. and Dean R.T. (1986) Fragmentation of proteins by free radicals and its effect on their susceptibility to enzymic hydrolysis. Biochem. J. 234, 399–403 10.1042/bj2340399 3718475 PMC1146578

[61] Davies K.J. (1985) Free radicals and protein degradation in human red blood cells. Prog. Clin. Biol. Res. 195, 15–27 3840596

[62] Dunlop R.A., Brunk U.T. and Rodgers K.J. (2009) Oxidized proteins: mechanisms of removal and consequences of accumulation. IUBMB Life 61, 522–527 10.1002/iub.189 19391165

[63] Grune T., Reinheckel T. and Davies K.J.A. (1997) Degradation of oxidized proteins in mammalian cells. FASEB J. 11, 526–534 10.1096/fasebj.11.7.9212076 9212076

[64] Mary J., Vougier S., Picot C.R., Perichon M., Petropoulos I. and Friguet B. (2004) Enzymatic reactions involved in the repair of oxidized proteins. Exp. Gerontol. 39, 1117–1123 10.1016/j.exger.2004.06.008 15359468

[65] Friguet B. (2006) Oxidized protein degradation and repair in ageing and oxidative stress. FEBS Lett. 580, 2910–2916 10.1016/j.febslet.2006.03.028 16574110

[66] Grant A.J., Jessup W. and Dean R.T. (1993) Inefficient degradation of oxidized regions of protein molecules. Free Radic. Res. Commun. 18, 259–267 10.3109/10715769309147493 8370549

[67] Grant A.J., Jessup W. and Dean R.T. (1992) Accelerated endocytosis and incomplete catabolism of radical-damaged protein. Biochim. Biophys. Acta - Mol. Cell Res. 1134, 203–209 10.1016/0167-4889(92)90177-D 1558844

[68] Davies K.J., Lin S.W. and Pacifici R.E. (1987) Protein damage and degradation by oxygen radicals. IV. Degradation of denatured protein. J. Biol. Chem. 262, 9914–9920 10.1016/S0021-9258(18)48021-0 3036878

[69] Pickering A.M. and Davies K.J.A. (2012) Degradation of damaged proteins. Prog. Mol. Biol. Transl. Sci. 109, 227–248 10.1016/B978-0-12-397863-9.00006-7 22727423 PMC3710712

[70] Dean R.T., Dunlop R., Hume P. and Rodgers K.J. (2003) Proteolytic “defences” and the accumulation of oxidized polypeptides in cataractogenesis and atherogenesis. Biochem. Soc. Symp. 70, 135–146, (Saklatvala, J., Nagase, H., and Salvesen, G., eds.) 10.1042/bss0700135 14587289

[71] Zhuang J., Chen X., Cai G., Wu D., Tu C., Zhu S. et al. (2021) Age-related accumulation of advanced oxidation protein products promotes osteoclastogenesis through disruption of redox homeostasis. Cell Death Dis. 12, 1160 10.1038/s41419-021-04441-w 34907153 PMC8671415

[72] Stadtman E.R., Starke-Reed P.E., Oliver C.N., Carney J.M. and Floyd R.A. (1992) Protein modification in aging. Free Radicals and Aging, pp. 64–72, Birkhäuser Basel, Basel 10.1007/978-3-0348-7460-1_7 1360283

[73] Nowotny K., Jung T., Grune T. and Höhn A. (2014) Accumulation of modified proteins and aggregate formation in aging. Exp. Gerontol. 57, 122–131 10.1016/j.exger.2014.05.016 24877899

[74] Brunk U.T. and Terman A. (2002) Lipofuscin: mechanisms of age-related accumulation and influence on cell function. Free Radic. Biol. Med. 33, 611–619 10.1016/S0891-5849(02)00959-0 12208347

[75] Kurz T., Terman A., Gustafsson B. and Brunk U.T. (2008) Lysosomes and oxidative stress in aging and apoptosis. Biochim. Biophys. Acta - Gen. Subj. 1780, 1291–1303 10.1016/j.bbagen.2008.01.009 18255041

[76] Ferrer-Sueta G., Campolo N., Trujillo M., Bartesaghi S., Carballal S., Romero N. et al. (2018) Biochemistry of peroxynitrite and protein tyrosine nitration. Chem. Rev. 118, 1338–1408 10.1021/acs.chemrev.7b00568 29400454

[77] Hartsema E.A., Hemmling H. and Hawkins C.L. (2025) Comparative reactivity of hypohalous acids with proteins: chemistry, biological effects and consequences. Adv. Redox Res. 14, 100119 10.1016/j.arres.2025.100119

[78] Hawkins C.L. (2020) Hypochlorous acid-mediated modification of proteins and its consequences. Essays Biochem. 64, 75–86 10.1042/EBC20190045 31867603

[79] Hunter E.P.L., Desrosiers M.F. and Simic M.G. (1989) The effect of oxygen, antioxidants, and superoxide radical on tyrosine phenoxyl radical dimerization. Free Radic. Biol. Med. 6, 581–585 10.1016/0891-5849(89)90064-6 2546863

[80] Fuentes-Lemus E., Hägglund P., López-Alarcón C. and Davies M.J. (2021) Oxidative crosslinking of peptides and proteins: mechanisms of formation, detection, characterization and quantification. Molecules 27, 15 10.3390/molecules27010015 35011250 PMC8746199

[81] Medinas D.B., Gozzo F.C., Santos L.F.A., Iglesias A.H. and Augusto O. (2010) A ditryptophan cross-link is responsible for the covalent dimerization of human superoxide dismutase 1 during its bicarbonate-dependent peroxidase activity. Free Radic. Biol. Med. 49, 1046–1053 10.1016/j.freeradbiomed.2010.06.018 20600836

[82] Rossi C., Fuentes-Lemus E. and Davies M.J. (2022) Reaction of cysteine residues with oxidized tyrosine residues mediates cross-linking of photo-oxidized casein proteins. Food Chem. 385, 132667 10.1016/j.foodchem.2022.132667 35299016

[83] Chellan P. and Nagaraj R.H. (1999) Protein crosslinking by the maillard reaction: dicarbonyl-derived imidazolium crosslinks in aging and diabetes. Arch. Biochem. Biophys. 368, 98–104 10.1006/abbi.1999.1291 10415116

[84] Richardson D.E., Yao H., Frank K.M. and Bennett D.A. (2000) Equilibria, kinetics, and mechanism in the bicarbonate activation of hydrogen peroxide: oxidation of sulfides by peroxymonocarbonate. J. Am. Chem. Soc. 122, 1729–1739 10.1021/ja9927467

[85] Alvarez B. and Radi R. (2003) Peroxynitrite reactivity with amino acids and proteins. Amino Acids 25, 295–311 10.1007/s00726-003-0018-8 14661092

[86] Tien M., Berlett B.S., Levine R.L., Chock P.B. and Stadtman E.R. (1999) Peroxynitrite-mediated modification of proteins at physiological carbon dioxide concentration: pH dependence of carbonyl formation, tyrosine nitration, and methionine oxidation. Proc. Natl. Acad. Sci. U.S.A. 96, 7809–7814 10.1073/pnas.96.14.7809 10393903 PMC22143

[87] Ellis R.J. (2001) Macromolecular crowding: an important but neglected aspect of the intracellular environment. Curr. Opin. Struct. Biol. 11, 114–119 10.1016/S0959-440X(00)00172-X 11179900

[88] Ellis R.J. (2001) Macromolecular crowding: obvious but underappreciated. Trends Biochem. Sci 26, 597–604 10.1016/S0968-0004(01)01938-7 11590012

[89] Rivas G. and Minton A.P. (2016) Macromolecular crowding in vitro, in vivo, and in between. Trends Biochem. Sci 41, 970–981 10.1016/j.tibs.2016.08.013 27669651 PMC5804487

[90] Monterroso B., Margolin W., Boersma A.J., Rivas G., Poolman B. and Zorrilla S. (2024) Macromolecular crowding, phase separation, and homeostasis in the orchestration of bacterial cellular functions. Chem. Rev. 124, 1899–1949 10.1021/acs.chemrev.3c00622 38331392 PMC10906006

[91] Pronk S., Lindahl E. and Kasson P.M. (2014) Dynamic heterogeneity controls diffusion and viscosity near biological interfaces. Nat. Commun. 5, 3034 10.1038/ncomms4034 24398864 PMC3971065

[92] Zhou B.-R., Liang Y., Du F., Zhou Z. and Chen J. (2004) Mixed macromolecular crowding accelerates the oxidative refolding of reduced, denatured lysozyme. J. Biol. Chem. 279, 55109–55116 10.1074/jbc.M409086200 15494409

[93] van den Berg B. (1999) Effects of macromolecular crowding on protein folding and aggregation. EMBO J. 18, 6927–6933 10.1093/emboj/18.24.6927 10601015 PMC1171756

[94] Guseman A.J., Perez Goncalves G.M., Speer S.L., Young G.B. and Pielak G.J. (2018) Protein shape modulates crowding effects. Proc. Natl. Acad. Sci. U.S.A. 115, 10965–10970 10.1073/pnas.1810054115 30301792 PMC6205421

[95] Martín I., Celaya G., Alfonso C., Moro F., Rivas G. and Muga A. (2014) Crowding activates ClpB and enhances its association with DnaK for efficient protein aggregate reactivation. Biophys. J. 106, 2017–2027 10.1016/j.bpj.2014.03.042 24806934 PMC4017315

[96] Stagg L., Zhang S.-Q., Cheung M.S. and Wittung-Stafshede P. (2007) Molecular crowding enhances native structure and stability of α/β protein flavodoxin. Proc. Natl. Acad. Sci. U.S.A. 104, 18976–18981 10.1073/pnas.0705127104 18024596 PMC2141893

[97] Qin S. and Zhou H.-X. (2009) Atomistic modeling of macromolecular crowding predicts modest increases in protein folding and binding stability. Biophys. J. 97, 12–19 10.1016/j.bpj.2009.03.066 19580740 PMC2711389

[98] Tsao D. and Dokholyan N.V. (2010) Macromolecular crowding induces polypeptide compaction and decreases folding cooperativity. Phys. Chem. Chem. Phys. 12, 3491–3500 10.1039/b924236h 20355290 PMC3050011

[99] White D.A., Buell A.K., Knowles T.P.J., Welland M.E. and Dobson C.M. (2010) Protein aggregation in crowded environments. J. Am. Chem. Soc. 132, 5170–5175 10.1021/ja909997e 20334356

[100] Jing W., Qin Y. and Tong J. (2020) Effects of macromolecular crowding on the folding and aggregation of glycosylated MUC5AC. Biochem. Biophys. Res. Commun. 529, 984–990 10.1016/j.bbrc.2020.06.156 32819609

[101] Bazmi S., Seifi B. and Wallin S. (2023) Simulations of a protein fold switch reveal crowding-induced population shifts driven by disordered regions. Commun. Chem. 6, 191 10.1038/s42004-023-00995-2 37689829 PMC10492864

[102] Zhou Y.-L., Liao J.-M., Chen J. and Liang Y. (2006) Macromolecular crowding enhances the binding of superoxide dismutase to xanthine oxidase: Implications for protein-protein interactions in intracellular environments. Int. J. Biochem. Cell Biol. 38, 1986–1994 10.1016/j.biocel.2006.05.012 16857407

[103] Minton A.P. and Wilf J. (1981) Effect of macromolecular crowding upon the structure and function of an enzyme: glyceraldehyde-3-phosphate dehydrogenase. Biochemistry 20, 4821–4826 10.1021/bi00520a003 7295652

[104] Wilcox X.E., Ariola A., Jackson J.R. and Slade K.M. (2020) Overlap concentration and the effect of macromolecular crowding on citrate synthase activity. Biochemistry 59, 1737–1746 10.1021/acs.biochem.0c00073 32216302

[105] Rastogi H. and Chowdhury P.K. (2021) Understanding enzyme behavior in a crowded scenario through modulation in activity, conformation and dynamics. Biochim. Biophys. Acta - Proteins Proteomics 1869, 140699 10.1016/j.bbapap.2021.140699 34298166

[106] Balcells C., Pastor I., Vilaseca E., Madurga S., Cascante M. and Mas F. (2014) Macromolecular crowding effect upon in vitro enzyme kinetics: Mixed activation-diffusion control of the oxidation of NADH by pyruvate catalyzed by lactate dehydrogenase. J. Phys. Chem. B 118, 4062–4068 10.1021/jp4118858 24660904

[107] Baumann P., Spulber M., Fischer O., Car A. and Meier W. (2017) Investigation of horseradish peroxidase kinetics in an “organelle-like” environment. Small 13, 1603943 10.1002/smll.201603943 28244215

[108] Sadarangani V., Kalia A., Kausar T., Murarka P. and Sau A.K. (2023) Effect of the macromolecular crowding agents on the structure and function of human arginase-I, a therapeutically important enzyme. J. Phys. Chem. B 127, 8749–8761 10.1021/acs.jpcb.3c02940 37796726

[109] Niki E. (1987) Lipid antioxidants: how they may act in biological systems. Br. J. Cancer Suppl. 8, 153–157 3307868 PMC2149475

[110] Fuentes-Lemus E., Jiang S., Hägglund P. and Davies M.J. (2021) High concentrations of casein proteins exacerbate radical chain reactions and increase the extent of oxidative damage. Food Hydrocoll. 121, 107060 10.1016/j.foodhyd.2021.107060

[111] Glover M.R., Davies M.J. and Fuentes-Lemus E. (2024) Oxidation of the active site cysteine residue of glyceraldehyde-3-phosphate dehydrogenase to the hyper-oxidized sulfonic acid form is favored under crowded conditions. Free Radic. Biol. Med. 212, 1–9 10.1016/j.freeradbiomed.2023.12.015 38122871

[112] Zhou H., Singh H., Parsons Z.D., Lewis S.M., Bhattacharya S., Seiner D.R. et al. (2011) The biological buffer bicarbonate/CO_2_ potentiates H_2_O_2_-mediated inactivation of protein tyrosine phosphatases. J. Am. Chem. Soc. 133, 15803–15805 10.1021/ja2077137 21913686 PMC3268130

[113] Fuentes-Lemus E., Reyes J.S., López-Alarcón C. and Davies M.J. (2022) Crowding modulates the glycation of plasma proteins: In vitro analysis of structural modifications to albumin and transferrin and identification of sites of modification. Free Radic. Biol. Med. 193, 551–566 10.1016/j.freeradbiomed.2022.10.319 36336230

[114] Perusko M., Al-Hanish A., Cirkovic Velickovic T. and Stanic-Vucinic D. (2015) Macromolecular crowding conditions enhance glycation and oxidation of whey proteins in ultrasound-induced Maillard reaction. Food Chem. 177, 248–257 10.1016/j.foodchem.2015.01.042 25660883

[115] Dargasz M., Anthuparambil N. Das, Retzbach S., Girelli A., Timmermann S., Möller J. et al. (2025) Depletion-induced interactions modulate nanoscale protein diffusion in polymeric crowder solutions. 10.48550/arXiv.2509.04087 PMC13439079 42525517

[116] Marenduzzo D., Finan K. and Cook P.R. (2006) The depletion attraction: an underappreciated force driving cellular organization. J. Cell Biol. 175, 681–686 10.1083/jcb.200609066 17145959 PMC2064666

[117] Ray S., Mason T.O., Boyens-Thiele L., Farzadfard A., Larsen J.A., Norrild R.K. et al. (2023) Mass photometric detection and quantification of nanoscale α-synuclein phase separation. Nat. Chem. 15, 1306–1316 10.1038/s41557-023-01244-8 37337111

[118] Vazquez D.S., Toledo P.L., Gianotti A.R. and Ermácora M.R. (2022) Protein conformation and biomolecular condensates. Curr. Res. Struct. Biol. 4, 285–307 10.1016/j.crstbi.2022.09.004 36164646 PMC9508354

[119] Bullier-Marchandin E., Gence D., Lamy H., Hervieu P., Ladam G.D., Thormann E. et al. (2025) Crowder-induced protein condensation: Role of the polymer concentration and mesh size in crowded systems. ACS Appl. Eng. Mater. 3, 1166–1176 10.1021/acsaenm.4c00802

[120] Villegas J.A., Heidenreich M. and Levy E.D. (2022) Molecular and environmental determinants of biomolecular condensate formation. Nat. Chem. Biol. 18, 1319–1329 10.1038/s41589-022-01175-4 36400992

[121] Oh H.J., Lee Y., Hwang H., Hong K., Choi H., Kang J.Y. et al. (2025) Size-controlled assembly of phase separated protein condensates with interfacial protein cages. Nat. Commun. 16, 1009 10.1038/s41467-025-56391-y 39856105 PMC11760349

[122] Yuzu K., Lin C.-Y., Yi P.-W., Huang C.-H., Masuhara H. and Chatani E. (2024) Spatiotemporal formation of a single liquid-like condensate and amyloid fibrils of α-synuclein by optical trapping at solution surface. Proc. Natl. Acad. Sci. U.S.A. 121, e2402162121 10.1073/pnas.2402162121 39292741 PMC11441557

[123] Bullier-Marchandin E., Philipo S., Marquis V., Echalard A., Ladam G. and Lutzweiler G. (2023) Investigation of the formation and aging of albumin-based condensates. ACS Appl. Eng. Mater. 1, 1634–1643 10.1021/acsaenm.3c00154

[124] Robles-Ramos M.Á., Zorrilla S., Alfonso C., Margolin W., Rivas G. and Monterroso B. (2021) Assembly of bacterial cell division protein FtsZ into dynamic biomolecular condensates. Biochim. Biophys. Acta - Mol. Cell Res. 1868, 118986 10.1016/j.bbamcr.2021.118986 33581219 PMC8529516

[125] Tian Z. and Qian F. (2021) Adenosine triphosphate-induced rapid liquid-liquid phase separation of a model IgG1 mAb. Mol. Pharm. 18, 267–274 10.1021/acs.molpharmaceut.0c00905 33307701

[126] Kang J., Lim L. and Song J. (2018) ATP enhances at low concentrations but dissolves at high concentrations liquid-liquid phase separation (LLPS) of ALS/FTD-causing FUS. Biochem. Biophys. Res. Commun. 504, 545–551 10.1016/j.bbrc.2018.09.014 30205960

[127] Dang M., Lim L., Kang J. and Song J. (2021) ATP biphasically modulates LLPS of TDP-43 PLD by specifically binding arginine residues. Commun. Biol. 4, 714 10.1038/s42003-021-02247-2 34112944 PMC8192790

[128] Hautke A. and Ebbinghaus S. (2023) The emerging role of ATP as a cosolute for biomolecular processes. Biol. Chem. 404, 897–908 10.1515/hsz-2023-0202 37656203

[129] Huang S., Xu B. and Liu Y. (2022) Calcium promotes α-synuclein liquid-liquid phase separation to accelerate amyloid aggregation. Biochem. Biophys. Res. Commun. 603, 13–20 10.1016/j.bbrc.2022.02.097 35276458

[130] Zheng Q., Chen Y., Chen D., Zhao H., Feng Y., Meng Q. et al. (2022) Calcium transients on the ER surface trigger liquid-liquid phase separation of FIP200 to specify autophagosome initiation sites. Cell 185, 4082.e22–4098.e22 10.1016/j.cell.2022.09.001 36198318

[131] Webb B.A., Dosey A.M., Wittmann T., Kollman J.M. and Barber D.L. (2017) The glycolytic enzyme phosphofructokinase-1 assembles into filaments. J. Cell Biol. 216, 2305–2313 10.1083/jcb.201701084 28646105 PMC5551713

[132] Kato M., Yang Y.-S., Sutter B.M., Wang Y., McKnight S.L. and Tu B.P. (2019) Redox state controls phase separation of the yeast Ataxin-2 protein via reversible oxidation of its methionine-rich low-complexity domain. Cell 177, 711.e8–721.e8 10.1016/j.cell.2019.02.044 30982603 PMC6752730

[133] Huang X., Chen S., Li W., Tang L., Zhang Y., Yang N. et al. (2021) ROS regulated reversible protein phase separation synchronizes plant flowering. Nat. Chem. Biol. 17, 549–557 10.1038/s41589-021-00739-0 33633378

[134] Pérez-Sala D. and Zorrilla S. (2025) Versatility of vimentin assemblies: from filaments to biomolecular condensates and back. Eur. J. Cell Biol. 104, 151487 10.1016/j.ejcb.2025.151487 40194320

[135] Martínez-Cenalmor P., Martínez A.E., Moneo-Corcuera D., González-Jiménez P. and Pérez-Sala D. (2024) Oxidative stress elicits the remodeling of vimentin filaments into biomolecular condensates. Redox Biol. 75, 103282 10.1016/j.redox.2024.103282 39079387 PMC11338992

[136] Zavaliev R., Mohan R., Chen T. and Dong X. (2020) Formation of NPR1 condensates promotes cell survival during the plant immune response. Cell 182, 1093.e18–1108.e18 10.1016/j.cell.2020.07.016 32810437 PMC7484032

[137] Harami G.M., Pálinkás J., Kovács Z.J., Jezsó B., Tárnok K., Harami-Papp H. et al. (2024) Redox-dependent condensation and cytoplasmic granulation by human ssDNA-binding protein-1 delineate roles in oxidative stress response. iScience 27, 110788 10.1016/j.isci.2024.110788 39286502 PMC11403420

[138] Vignane T., Hugo M., Hoffmann C., Katsouda A., Petric J., Wang H. et al. Protein thiol alterations drive aberrant phase separation in aging. bioRxiv 10.1101/2023.11.07.566021 PMC13468144 42533105

[139] Vedula P., Ishizuka K., Hayashida A., Sueo K. and Sawa A. (2025) Stress-induced nuclear GAPDH: scientific update and clinical application. Neurotherapeutics 22, e00725 40903341 10.1016/j.neurot.2025.e00725 PMC12664512

[140] Shi M., Hou J., Liang W., Li Q., Shao S., Ci S. et al. (2023) GAPDH facilitates homologous recombination repair by stabilizing RAD51 in an HDAC1‐dependent manner. EMBO Rep. 24 EMBR202256437 10.15252/embr.202256437 PMC10398663 37306047

[141] Mustafa Rizvi S.H., Shao D., Tsukahara Y., Pimentel D.R., Weisbrod R.M., Hamburg N.M. et al. (2021) Oxidized GAPDH transfers S-glutathionylation to a nuclear protein Sirtuin-1 leading to apoptosis. Free Radic. Biol. Med. 174, 73–83 10.1016/j.freeradbiomed.2021.07.037 34332079 PMC8432375

[142] Uechi H., Sridharan S., Nijssen J., Bilstein J., Iglesias-Artola J.M., Kishigami S. et al. (2025) Small-molecule dissolution of stress granules by redox modulation benefits ALS models. Nat. Chem. Biol. 21, 1577–1588 10.1038/s41589-025-01893-5 40369342 PMC12463676

[143] Gordon P.M., Hamid F., Makeyev E.V. and Houart C. (2021) A conserved role for the ALS-linked splicing factor SFPQ in repression of pathogenic cryptic last exons. Nat. Commun. 12, 1918 10.1038/s41467-021-22098-z 33771997 PMC7997972

[144] Filipovic M.R. (2025) Profiling the landscape of cysteine posttranslational modifications in brain aging and neurodegeneration. Neurotherapeutics 22, e00726 10.1016/j.neurot.2025.e00726 40887391 PMC12664503

[145] Murphy M.P., Bayir H., Belousov V., Chang C.J., Davies K.J.A., Davies M.J. et al. (2022) Guidelines for measuring reactive oxygen species and oxidative damage in cells and in vivo. Nat. Metab. 4, 651–662 10.1038/s42255-022-00591-z 35760871 PMC9711940

[146] Christiansen A. and Wittung-Stafshede P. (2013) Quantification of excluded volume effects on the folding landscape of Pseudomonas aeruginosa Apoazurin in vitro. Biophys. J. 105, 1689–1699 10.1016/j.bpj.2013.08.038 24094410 PMC3791299

[147] Biswas S., Kundu J., Mukherjee S.K. and Chowdhury P.K. (2018) Mixed macromolecular crowding: a protein and solvent perspective. ACS Omega 3, 4316–4330 10.1021/acsomega.7b01864 30023892 PMC6044960

[148] Fuentes-Lemus E., Reyes J.S., Gamon L.F., López-Alarcón C. and Davies M.J. (2021) Effect of macromolecular crowding on protein oxidation: Consequences on the rate, extent and oxidation pathways. Redox Biol. 48, 102202 10.1016/j.redox.2021.102202 34856437 PMC8640551

[149] Hawkins C.L. and Davies M.J. (2019) Detection, identification, and quantification of oxidative protein modifications. J. Biol. Chem. 294, 19683–19708 10.1074/jbc.REV119.006217 31672919 PMC6926449

[150] Hawkins C.L., Morgan P.E. and Davies M.J. (2009) Quantification of protein modification by oxidants. Free Radic. Biol. Med. 46, 965–988 10.1016/j.freeradbiomed.2009.01.007 19439229

[151] Kisty E.A., Falco J.A. and Weerapana E. (2023) Redox proteomics combined with proximity labeling enables monitoring of localized cysteine oxidation in cells. Cell Chem. Biol. 30, 321.e6–336.e6 10.1016/j.chembiol.2023.02.006 36889310 PMC10069010

[152] Potekhina E.S., Bass D.I., Ezeriņa D., Fleckenstein D.D., Chebotarev A.S., Sysoeva V.A. et al. (2025) A color-tailored fluorogenic sensor for hydrogen peroxide. Nat. Chem. Biol. 22 568–579 41102408 10.1038/s41589-025-02036-6

[153] Kritsiligkou P., Bosch K., Shen T.K., Meurer M., Knop M. and Dick T.P. (2023) Proteome-wide tagging with an H_2_O_2_ biosensor reveals highly localized and dynamic redox microenvironments. Proc. Natl. Acad. Sci. U.S.A. 120, e2314043120 10.1073/pnas.2314043120 37991942 PMC10691247

[154] Grassmann G., Miotto M., Desantis F., Di Rienzo L., Tartaglia G.G., Pastore A. et al. (2024) Computational approaches to predict protein-protein interactions in crowded cellular environments. Chem. Rev. 124, 3932–3977 10.1021/acs.chemrev.3c00550 38535831 PMC11009965

[155] Bolduc J., Koruza K., Luo T., Malo Pueyo J., Vo T.N., Ezeriņa D. et al. (2021) Peroxiredoxins wear many hats: factors that fashion their peroxide sensing personalities. Redox Biol. 42, 101959 10.1016/j.redox.2021.101959 33895094 PMC8113037

